# Fluorinated phenylalanines: synthesis and pharmaceutical applications

**DOI:** 10.3762/bjoc.16.91

**Published:** 2020-05-15

**Authors:** Laila Fathy Awad, Mohammed Salah Ayoup

**Affiliations:** 1 Chemistry Department, Faculty of Science, Alexandria University, P.O. Box 426, Alexandria, 21321, Egypt

**Keywords:** α-fluorophenylalanine, β- and β,β-difluorophenylalanine, fluorinated phenylalanines, PET, pharmaceuticals application

## Abstract

Recent advances in the chemistry of peptides containing fluorinated phenylalanines (Phe) represents a hot topic in drug research over the last few decades. ᴅ- or ʟ-fluorinated phenylalanines have had considerable industrial and pharmaceutical applications and they have been expanded also to play an important role as potential enzyme inhibitors as well as therapeutic agents and topography imaging of tumor ecosystems using PET. Incorporation of fluorinated aromatic amino acids into proteins increases their catabolic stability especially in therapeutic proteins and peptide-based vaccines. This review seeks to summarize the different synthetic approaches in the literature to prepare ᴅ- or ʟ-fluorinated phenylalanines and their pharmaceutical applications with a focus on published synthetic methods that introduce fluorine into the phenyl, the β-carbon or the α-carbon of ᴅ-or ʟ-phenylalanines.

## Introduction

Major efforts have been focused on the synthesis of fluorinated organic molecules particularly for drug development. The replacement of hydrogen by fluorine has been used in the development to improve the biophysical and chemical properties of bioactives. Such tuning in properties arises from the small size of fluorine, the next in size to hydrogen. However, the high electronegativity of the fluorine leads to low polarizability and a strong covalent bond to carbon [1–4]. Therefore, the introduction of fluorine into phenylalanine (Phe) can modulate the acidity, basicity, hydrophobicity, geometry, conformation, reactivity, and moreover the bioavailability of the analogue [1]. Fluorinated amino acids (FAAs) have considerable industrial and pharmaceutical potential [2]. Also, they have played an important role as enzyme inhibitors as well as therapeutic agents [3–4]. Moreover, they modulate the properties of peptides and proteins [5–7], influencing aspects such as protein folding, protein–protein interactions, ribosomal translation, lipophilicity, acidity/basicity, optimal pH, stability, thermal stability, and therapeutic properties [8–10]. This extends to metabolic properties of membrane permeability and reactivity [11–14]. The effect of peptide structure and stability has been found to depend on the position and number of fluorine atoms within the amino acid chains [15–17]. Incorporation of fluorinated aromatic amino acids into proteins can increase their shelf life, especially in therapeutic proteins and peptide-based vaccines [18]. Enhanced catabolic stability [6] can arise from the role of particular aromatic amino acids in membrane–protein interactions [19]. Furthermore, fluorinated aromatic amino acids can alter enzymatic activity as a result of enhanced protein stability [5]. Also fluorinated aromatic amino acids can destabilize ΙΙ-cation interactions whereas their increased hydrophobicity enhances binding affinity [19]. Moreover, the incorporation of fluorinated amino acids into proteins provides the opportunity for probing structure (by NMR techniques) including protein–protein and protein–ligand interactions and consequently metabolic processes [20–21].

Fluorinated phenylalanines (FPhe) have been incorporated into various proteins and enzymes [22–25] with advantageous biophysical, chemical, and biological properties, and their effect on the stability and activity of peptides in therapeutic vaccines and enzymes has been studied [19,26–33].

**Figure 1:**
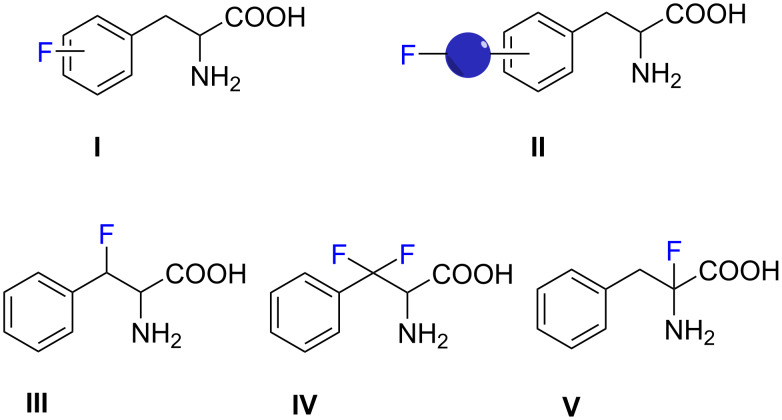
Categories **I**–**V** of fluorinated phenylalanines.

In this review we provide an overview for the various syntheses of FPhes and analogues. Five different categories of FPhe are represented and are classified **I**–**V** according to the position of the fluorine(s) (Figure 1).

## Review

### 1. Synthesis of fluorinated phenylalanine of type **I** and **II**

Direct attachment of the fluorine atom to the aryl ring of Phe or fluorinated groups directly attached to a spacer extending from the aryl ring constitute types **I** and **II** (Figure 1), accordingly we reported herein different methods for their synthesis.

#### 1.1. Negishi cross coupling of aryl halide and organozinc compounds

Jackson and co-workers reported the synthesis of a range of phenylalanine derivatives via Negishi cross-coupling reactions of aryl halides and Zn homoenolates of the protected (*R*)-iodoalanine **2**. The reaction was activated using Pd(0) as a catalyst.

**Scheme 1:**
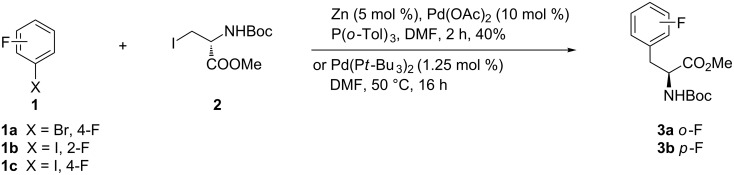
Synthesis of fluorinated phenylalanines via Jackson’s method.

A palladium-catalyzed cross-coupling reaction between an organozinc iodide and aryl halides offers a convenient method for the direct preparation of protected fluorinated Phe analogues **3**. Thus, cross coupling of the protected iodoalanine **2** with 4-fluorobromobenzene (**1a**) or 2- and 4-fluoroiodobenzene (**1b** and **1c**), respectively, was accomplished using the reported coupling conditions in Scheme 1 to give the *N*-Boc-protected 2- or 4-fluoro-ʟ-phenylalanine esters **3a**,**b** [34–35].

On the other hand, attempted cross-coupling of fluoroiodobenzenes **1b** and **1c** with iodoalanine **2** at room temperature using Pd_2_(dba)_3_ (2.5 mol %) and SPhos (5.0 mol %) provided excellent yields of (*S*)-phenylalanines **3a** (70% yield) and **3b** (80% yield), respectively. Such an efficiency improvement testifies to the suitability of SPhos as a ligand for these coupling reactions, rather than the less-reactive organozinc reagents [36] (Scheme 1). Decreasing the molar ratio of Pd_2_(dba)_3_/SPhos to 0.25:0.5 mol % provided a lower yield of **3a** (21%), whereas **3b** showed only a slight decrease in yield (77%).

**Scheme 2:**
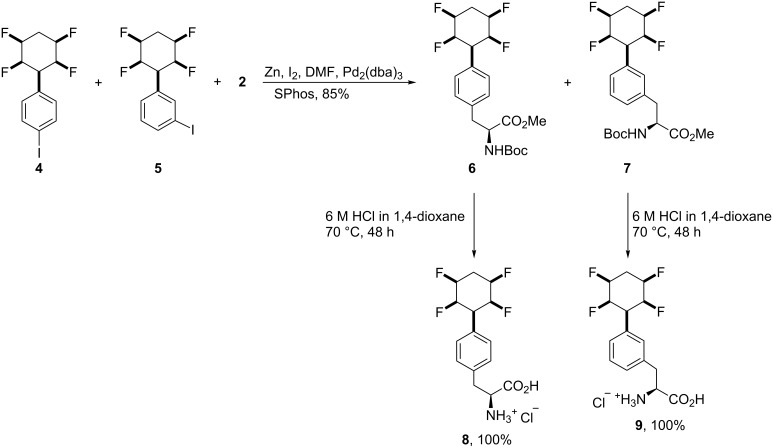
Synthesis of all-*cis*-tetrafluorocyclohexylphenylalanines.

The two-inseparable *para*/*meta* isomers of all-*cis*-2,3,5,6-tetrafluorocyclohexylphenyliodide **4** and **5** were subjected to Jackson’s methodology for the synthesis of the appropriate amino acid products. Thus, the coupling of the zinc homoenolate of (*R*)-iodoalanine **2** with a mixture of **4** and **5** in the presence of Pd(dba)_3_ and SPhos resulted in an excellent conversion to the fully protected amino acid isomers **6** and **7**, which were readily separated from each other by chromatography. Deprotection of isomers **6** and **7** gave the individual free amino acids *p-*(*S*)-**8** and *m-*(*S*)-**9** [37–38] (Scheme 2).

**Scheme 3:**
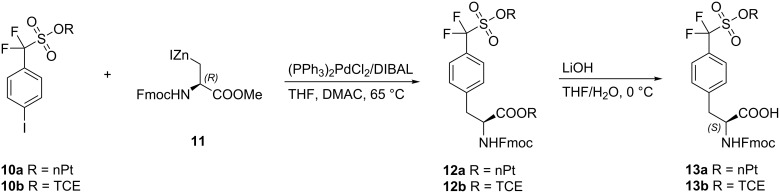
Synthesis of ʟ-4-[sulfono(difluoromethyl)]phenylalanine (nPt: neopentyl, TCE: trichloroethyl).

Coupling of one molar equivalent of neopentyl sulfonate ester **10a** or trichloroethyl ester **10b** with two molar equivalents of the zincate of Fmoc-3-iodo-ʟ-alanine methyl ester **11**, to form the protected ʟ-4-[sulfono(difluoromethyl)]phenylalanines **12a** and **12b**, was carried out using 5 mol % (PPh_3_)_2_PdCl_2_ and 10 mol % DIBAL in THF/DMAC 1:1 at 65–70 °C for 6 h. Partial deprotection by alkaline hydrolysis of **12a** and **12b** afforded ʟ-4-[sulfono(difluoromethyl)]phenylalanine derivatives **13a** and **13b**, respectively [39] (Scheme 3).

#### 1.2. Alkylations of fluorinated aryl halides with a chiral auxiliary

**Scheme 4:**
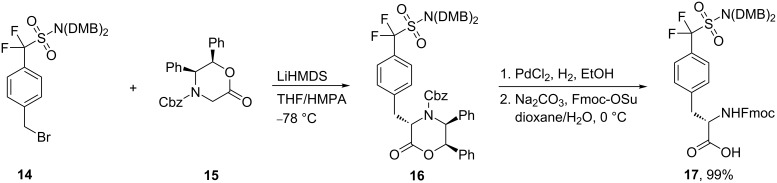
Synthesis of ʟ-4-[sulfono(difluoromethyl)]phenylalanine derivatives **17**.

Alternatively, the coupling of the bis(dimethoxybenzyl)-protected sulfonamide **14**, instead of the esters **10a** and **10b** with zincate **11** using a variety of catalysts and different reaction conditions, was unsuccessful. However, coupling of 4-[bis(dimethoxybenzyl)difluoromethyl]benzyl bromide (**14**) with the lithium enolate of William`s lactone **15** gave the protected amino acid **16** in 80% yield. The desired protected amino acid **17** was readily obtained after reduction of **16** using PdCl_2_ as a catalyst, followed by treating the product with Fmoc-OSu in dioxane/aq Na_2_CO_3_ (99%, two steps) [40] as illustrated in Scheme 4.

**Scheme 5:**
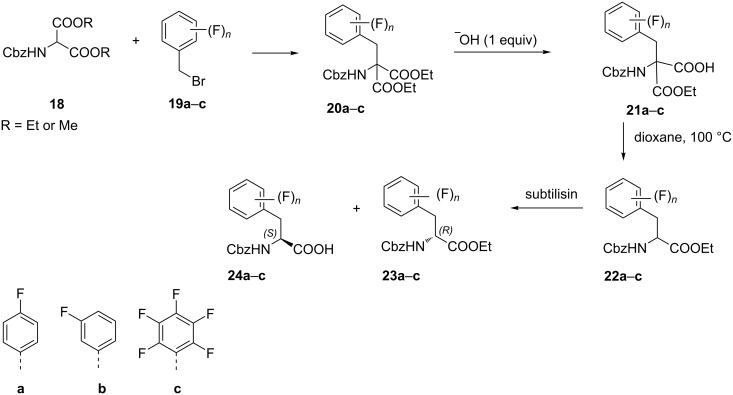
Synthesis of fluorinated Phe analogues from Cbz-protected aminomalonates.

The reaction of aminomalonate **18** with fluorinated benzyl bromides **19a–c** afforded the corresponding aralkyl diesters **20a–c**. Partial hydrolysis followed by decarboxylation gave the *N*-benzyloxycarbonyl ᴅʟ-amino acid esters **22a–c**, which upon enzymatic hydrolysis of the ester group using the subtilisin-type Carlsberg enzyme led to ᴅ-amino acid esters **23a–c** and the corresponding Cbz-protected *p*, *m*-fluoro-, or pentafluoro-ʟ-phenylalanine derivatives **24a**–**c**, respectively [41] (Scheme 5).

**Scheme 6:**
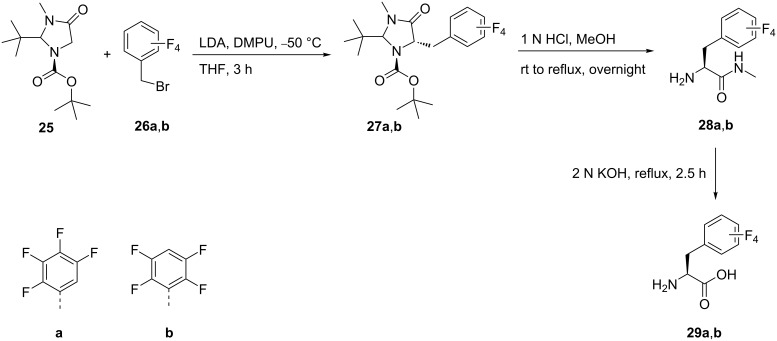
Synthesis of tetrafluorophenylalanine analogues via the 3-methyl-4-imidazolidinone auxiliary **25**.

The stereoselective benzylation of (*S*)*-*imidazolidinone ((*S*)-Boc-BMI) **25** with tetrafluorobenzyl bromides **26a**,**b** afforded the benzylated imidazolidinones **27a**,**b**. The acidic hydrolysis of **27a**,**b** with simultaneous deprotection to release the free amines gave amides **28a**,**b**. Treatment of the latter with aqueous potassium hydroxide finally afforded (*S*)-2-amino-3-(2,3,4,5-tetrafluorophenyl)propionic acid (**29a**) and (*S*)-2-amino-3-(2,3,5,6-tetrafluorophenyl)propionic acid (**29b**) [42] (Scheme 6).

**Scheme 7:**
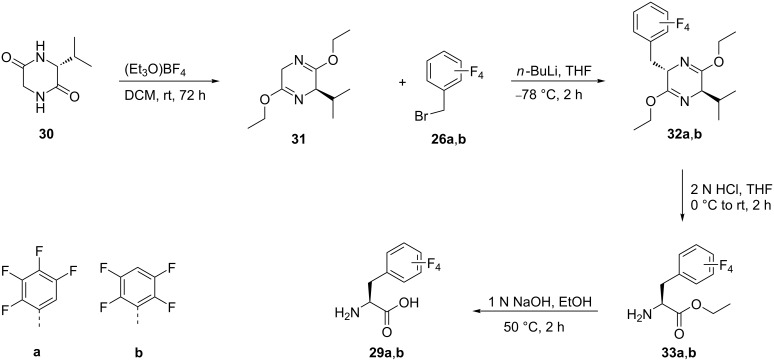
Synthesis of tetrafluoro-Phe derivatives via chiral auxiliary **31**.

Alternatively, phenylalanines **29a**,**b** were also synthesized by alkylation of **26a**,**b** with the chiral auxiliary **31**, which was obtained by reaction of the cyclic dipeptide **30** with triethyloxonium tetrafluoroborate. The alkylation reaction of **26a**,**b** was carried out with *n*-BuLi in THF at −78 °C to give **32a**,**b**. Acid hydrolysis of the alkylated product **32a**,**b** afforded the ethyl esters of tetrafluorophenylalanine **33a**,**b**, which by alkaline hydrolysis afforded the tetrafluoro derivatives **29a** and **29b**, respectively [43] (Scheme 7).

**Scheme 8:**
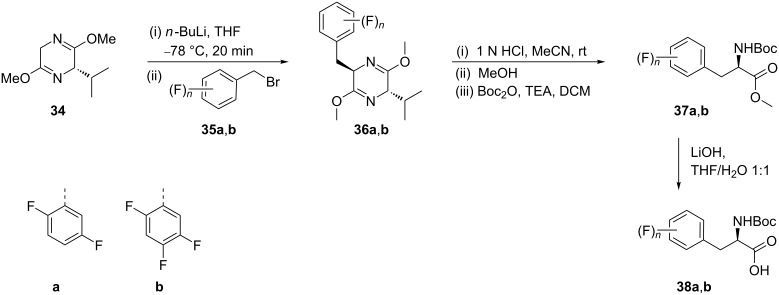
Synthesis of 2,5-difluoro-Phe and 2,4,5-trifluoro-Phe via Schöllkopf reagent **34**.

The syntheses of (*R*)-**38a** and (*R*)-**38b** were carried out by alkylation of the Schöllkopf reagent ((2*S*)-(+)-2,5-dihydro-3,6-dimethoxy-2-isopropylpyrazine, **34**) with the corresponding fluorinated benzyl bromides **35a**,**b** via intermediates **36a**,**b**. The alkylation products were then hydrolyzed to generate the amino acid esters which were directly Boc protected to give the *N*-Boc-protected amino acid methyl esters **37a**,**b**. Finally, ester hydrolysis afforded the useful Boc-(*R*)-amino acids **38a**,**b** [44] (Scheme 8).

**Scheme 9:**
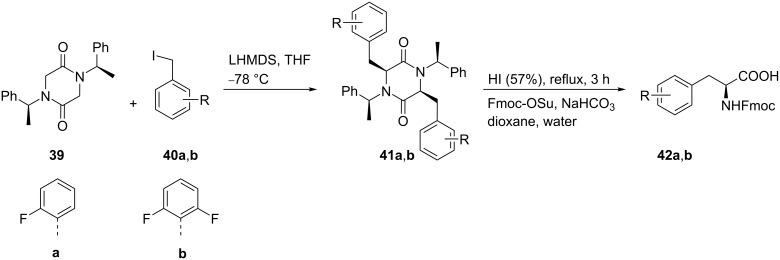
Synthesis of 2-fluoro- and 2,6-difluoro Fmoc-Phe derivatives starting from chiral auxiliary **39**.

A one-pot double alkylation of the chiral auxiliary **39** with benzyl iodides **40a**,**b** gave *cis*-dialkyl derivatives **41a**,**b** in 70–72% yield. The subsequent removal of the auxiliary followed by treatment with Fmoc-OSu gave the *N*-protected 2-fluoro- and 2,6-difluorophenylalanine derivatives **42a**,**b** in quantitative yields [45] (Scheme 9).

**Scheme 10:**
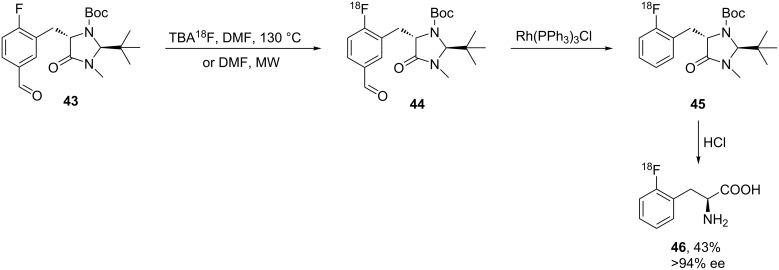
Synthesis of 2-[^18^F]FPhe via chiral auxiliary **43**.

The radiolabeled 2-[^18^F]-fluoro-ʟ-phenylalanine **46** was synthesized as a promising radiopharmaceutical agent for molecular imaging by positron emission tomography (PET). The three-step synthesis of **46** started from [^18^F]-fluoride exchange in **43** to generate **44**. The isotope exchange was explored by using [^18^F]-TBA in DMF at 130 °C for 10 min to give [^18^F]-**44**. Decarbonylation of **44** was achieved by treatment with Rh(PPh_3_)_3_Cl to afford **45** and the subsequent removal of protecting groups gave **46**. Conventional reactions yielded the desired product 2-[^18^F]FPhe **46** in 43% yield, whereas under microwave irradiation a 34% yield was obtained. Under the optimized conditions, the enantiomeric purity was reported to be ≥94% ee [46] (Scheme 10).

#### 1.3. Photooxidative cyanation of fluorinated benzylamine

**Scheme 11:**
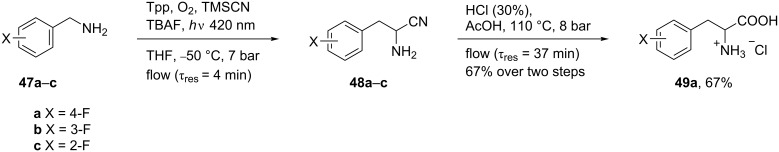
Synthesis of FPhe **49a** via photooxidative cyanation.

A convenient, protecting group-free, and semicontinuous process was reported for the synthesis of racemic fluorinated phenylalanine·HCl starting from benzylamines **47a–c**. Thus, a singlet oxygen-driven photooxidative cyanation of amines **47a–c** using tetraphenylporphyrin (Tpp), followed by an acid-mediated hydrolysis of the intermediate fluorinated α-amino nitrile **48a** with 30% HCl aq/acetic acid, gave the 4-fluorophenylalanine·HCl **49a** in a good overall yield (67%) [47] (Scheme 11).

#### 1.4. Hydrolysis of Erlenmeyer’s azalactone

**Scheme 12:**
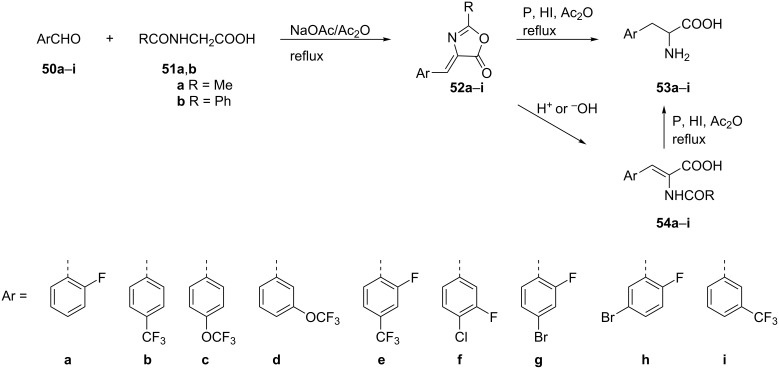
Synthesis of FPhe derivatives via Erlenmeyer azalactone synthesis.

A multistep Erlenmeyer azalactone synthesis was reported as an important method for the synthesis of fluorinated α-amino acids **53a–h**. Thus, a three-component condensation of a series of fluorinated benzaldehydes **50a–h**, *N*-acetyl- or *N*-benzoylglycine **51a** or **51b**, respectively, and an excess of acetic anhydride in the presence of sodium acetate afforded the oxazolones **52a–h**. The subsequent reductive ring cleavage of **52a–h** without isolation, was carried out with red phosphorus in hydroiodic acid to give the fluorinated phenylalanine analogues **53a–h**. Alternatively, a two-step sequence to generate amino acids **53a–h** was attempted by first hydrolysis of **52a–h** to form acids **54a–h** which then were reduced with P/HI to the desired products **53a–h** [48]. The free amino acid **53i** was prepared by the same protocol [49] (Scheme 12).

**Scheme 13:**
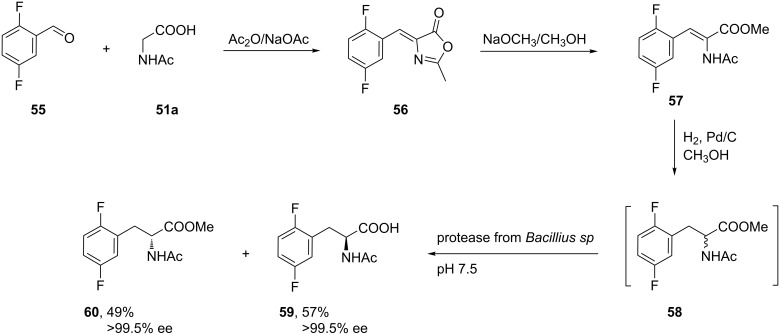
Synthesis of (*R*)- and (*S*)-2,5-difluoro Phe via the azalactone method.

2,5-Difluorophenylalanines with either *R* or *S* configuration were synthesized also via the Erlenmeyer azalactone method. The synthesis started with the multicomponent reaction of aldehyde **55**, acetylglycine **51a** and acetic anhydride to give the azalactone **56**. The subsequent basic hydrolysis of **56** gave **57** that, on catalytic hydrogenation, afforded racemic difluorinated Phe **58**. The isomers were separated by selective hydrolysis using a protease from *Bacillius sp* to generate the (*S*)-*N*-acetyl acid **59** with >99.5% ee and the corresponding (*R*)-*N*-acetyl ester **60** with >99.5% ee [50] (Scheme 13).

**Scheme 14:**
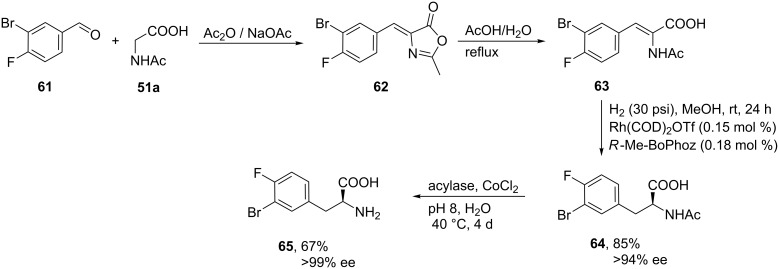
Synthesis of 3-bromo-4-fluoro-(*S*)-Phe (**65**).

The synthesis of 3-bromo-4-fluoro-(*S*)-Phe (**65**) was carried out by reacting 3-bromo-4-fluorobenzaldehyde (**61**) and *N*-acetylglycine (**51a**) to provide intermediate **63** in high yield and without purification. Then, the transition-metal-catalyzed asymmetric hydrogenation of the α-amidocinnamic acid **63** using the less frequently used ferrocene-based ligand Me-BoPhoz led to the *N*-acetyl-ʟ-phenylalanine derivative **64** with complete conversion and with 94% ee. The desired enantiomer (*S*)-**65** was obtained as a single isomer (>99% ee) after selective enzymatic hydrolysis of **64** using an acylase under mild conditions (pH 8.0, 40 °C, 4 d, with CoCl_2_ as co-factor) [51] (Scheme 14).

#### 1.5. Direct radiofluorination of ʟ-phenylalanine

**Scheme 15:**
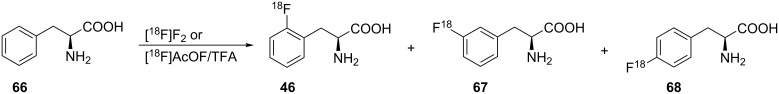
Synthesis of [^18^F]FPhe via radiofluorination of phenylalanine with [^18^F]F_2_ or [^18^F]AcOF.

The direct radiofluorination of ʟ-phenylalanine (**66**) with either [^18^F]F_2_ or [^18^F]AcOF in trifluoroacetic acid (TFA) afforded the three isomeric *o*, *m*, and *p*-fluoro-ʟ-phenylalanines **46**, **67**, and **68**, in ratio 72.5:13.9:13.6, respectively. In this reaction, [^18^F]AcOF showed a higher regioselectivity and less side product formation compared with [^18^F]F_2_ [52] (Scheme 15).

**Scheme 16:**
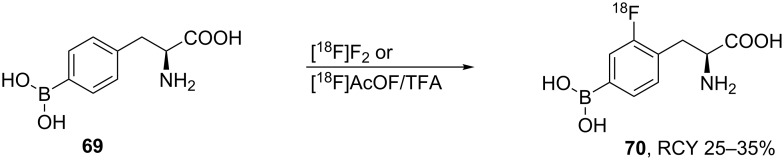
Synthesis of 4-borono-2-[^18^F]FPhe.

The radiolabeled compound, 4-borono-2-[^18^F]fluoro-ʟ-phenylalanine (**70**) was prepared by direct fluorination of 4-borono-ʟ-phenylalanine (BPA, **69**) with [^18^F]AcOF or [^18^F]F_2_. The reaction was followed by a HPLC separation using a Delta-Pak Cl8 cartridge 0.1% acetic acid as the mobile phase at a flow rate of 10 mL/min. The product was isolated in radiochemical yields of 25–35% and with a radiochemical purity of more than 99%. The ^18^F-labeling of 4-borono-ᴅ,ʟ-phenylalanine (BPA) provided a potential tool for cancer treatment by boron neutron capture therapy [53] (Scheme 16).

**Scheme 17:**
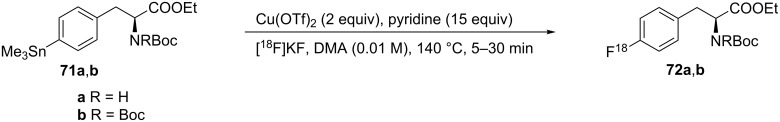
Synthesis of protected 4-[^18^F]FPhe via arylstannane derivatives.

The syntheses of a variety of clinically relevant radiotracers including protected 4-[^18^F]fluorophenylalanines **72a**,**b** [54] were achieved by a copper-mediated nucleophilic radiofluorination of arylstannanes **71a**,**b** with [^18^F]KF (Scheme 17).

#### 1.6. Alkylation of benzophenone imine of glycine ester

**Scheme 18:**
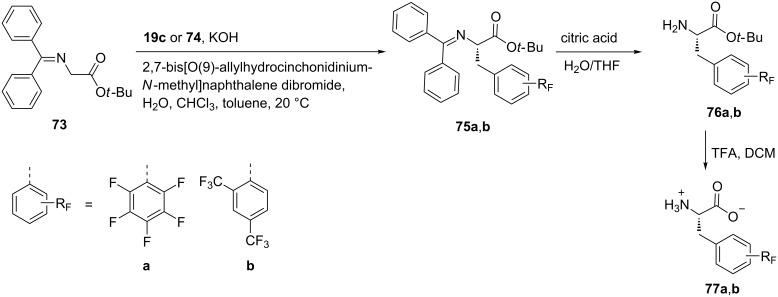
Synthesis of FPhe derivatives via intermediate imine formation.

Pentafluoro-ʟ-phenylalanine (**77a**) and 2,4-ditrifluoromethyl-ʟ-phenylalanine (**77b**) were synthesized through alkylation of the benzophenone imine of glycine ester **73**, with perfluorinated benzylbromide **19c** or 2,4-bis(trifluoromethyl)benzyl bromide (**74**) in the presence of 2,7-bis[*O*(9)-allylhydrocinchonidinium-*N*-methyl]naphthalene dibromide, to afford the fluorinated phenylalanine imines **75a**,**b** with ees < 98%. The products were hydrolyzed and deprotected in a two-step protocol to afford the desired products **77a**,**b** [55] (Scheme 18).

Interestingly substitution of Phe by either **77b** or **77a** in the proteasome inhibitors bortezomib or epoxymicin, led to an increase in the efficiency as anticancer proteasome inhibitors. The fluorinated amino acids **77a** and **77b** were used mainly for two reasons, i.e., the ready availability and hydrophobicity [55].

Further, (*S*)-pentafluorophenylalanine (Pff, **77a**) was used to stabilize proteins for potential applications in various protein-based biotechnologies. To improve protein stability, natural hydrocarbon amino acids were replaced with Pff **77a**. The effect of enhanced protein stability upon this replacement is referred as to ‘fluoro-stabilization effect’ [56].

#### 1.7. Knoevenagel condensation of methyl isocyanoacetate

**Scheme 19:**
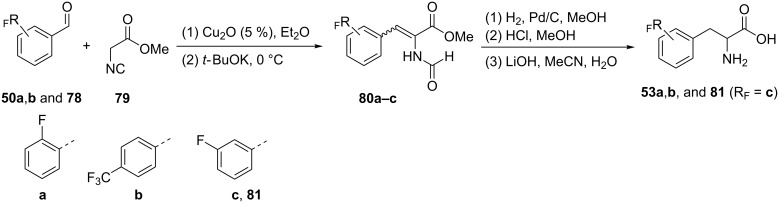
Synthesis of FPhe derivatives via Knoevenagel condensation.

Three isomers of fluorinated phenylalanines **53a**,**b** and **81** were synthesized by Knoevenagel condensation of methyl isocyanoacetate (**79**) and the corresponding fluorinated benzaldehyde derivatives **50a**,**b**, and *m*-fluorobenzaldehyde (**78**) as electrophiles in the presence of catalytic amounts of Cu(I) and base. The cinnamate derivatives **80a–c** obtained were hydrogenated either under homogeneous or heterogeneous conditions followed by deprotection of both the amide and the ester moieties to give the racemic fluorinated phenylalanines **53a**,**b**, or *m*-fluorophenylalanine (**81**) with good yields [57] (Scheme 19).

#### 1.8. Coupling of *N*-hydroxytetrachlorophthalimide esters with boronic acids

**Scheme 20:**
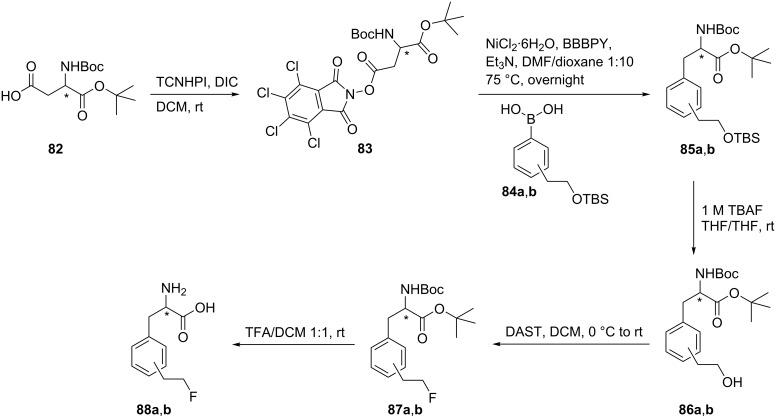
Synthesis of FPhe derivatives **88a**,**b** from aspartic acid derivatives.

4-(2-Fluoroethyl)-ʟ-phenylalanine and 3-(2-fluoroethyl)-ʟ-phenylalanine (**88a**,**b**), respectively, were synthesized starting from partially protected ʟ- or ᴅ-aspartic acid derivatives **82** which were activated as the *N*-hydroxytetrachlorophthalimide esters **83**. The treatment of the esters **83** with boronic acids **84a**,**b** afforded the substituted phenylalanine derivatives **85a**,**b**, respectively [58]. Deprotection of the hydroxy group was achieved by treatment with TBAF in THF to give **86a**,**b**. Finally, fluorination of the alcohols **86a**,**b** with DAST followed by deprotection gave the targeted compounds **88a**,**b** (Scheme 20).

**Scheme 21:**
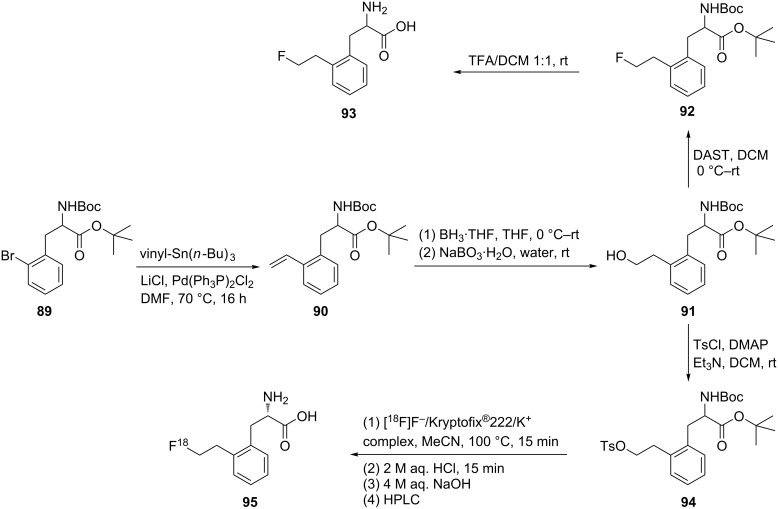
Synthesis of 2-(2-fluoroethyl)phenylalanine derivatives **93** and **95**.

Cross-coupling reactions with boronic acids were found to be successful only for the synthesis of *para* and *meta*-derivatives. Several attempts were made to prepare the *ortho*-substituted derivatives **93** and **95**. The synthesis of ᴅ,ʟ-**93** or ʟ-**95** was achieved by vinylation of the protected ᴅ,ʟ-*N*-Boc-2-bromophenylalanine (**89**) using a Stille coupling reaction to give the *o*-vinyl derivative **90** as key intermediate. A hydroboration reaction of compound **90** afforded the primary alcohol **91**, which was directly fluorinated and deprotected to give the free amino acids **93** (ᴅ and ʟ). Alternatively, alcohol **91** was activated by tosylation to give **94** as a precursor for radiofluorination that was achieved to give 2-[^18^F]FELP ʟ-**95** using [^18^F]-fluoride complexed with Kryptofix^®^/K^+^ followed by deprotection with HCl and purification. This product emerged as promising new PET tracer for brain tumor imaging [58] (Scheme 21).

#### 1.9. Alkylation and hydrolysis of Ni(II) or Zn(II) complexes

**Scheme 22:**
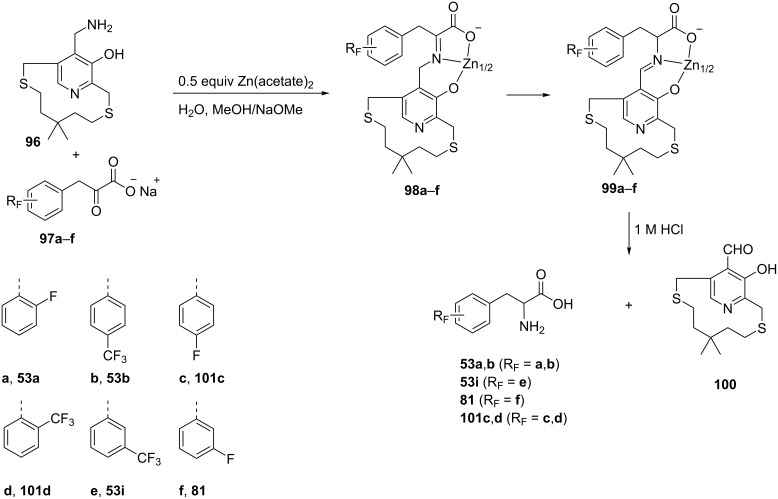
Synthesis of FPhe derivatives via Zn^2+^ complexes.

The synthesis of a series of fluorinated phenylalanines was achieved by transamination reactions between (*R*) or (*S*)-(15-aminomethyl-14-hydroxy-5,5-dimethyl-2,8-dithia[9](2,5)pyridinophanes) **96** and the sodium salt of *o*, *m*, or *p*-fluoro or (trifluoromethyl)phenylpyruvic acids **97a–f**. The reaction was carried out in presence of 0.5 equiv of zinc(II) acetate in the presence of NaOMe. The initially formed complexes **98a–f** underwent isomerization to **99a–f**. Acid hydrolysis then gave the FPhe derivatives **53a**,**b**, **53i**, **81**, and **101c**,**d** with modest enantiomeric excesses (33–66% ee) and in moderate yields [59] (Scheme 22).

**Scheme 23:**
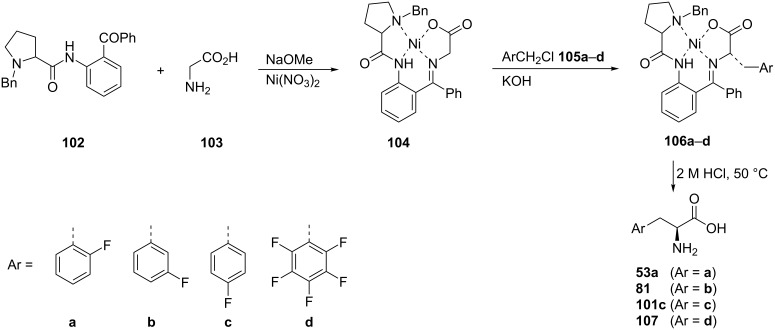
Synthesis of FPhe derivatives via Ni^2+^ complexes.

A convenient preparative method for the synthesis of enantiomerically pure *o*-, *m*-, and *p*- or pentafluorinated phenylalanines **53a**, **81**, **101c**, and **107** was carried out by the alkylation of glycine. The Ni(II) complex **104** was obtained through the reaction of **102** with glycine (**103**) and Ni(NO_3_)_2_. The subsequent alkylation of complex **104** with fluorine-containing benzyl chlorides **105a–d** followed by hydrolysis with HCl afforded enantiomerically enriched (<90% ee) (*S*)-fluorinated phenylalanine derivatives **53a**, **81**, **101c**, and **107** [60–61] (Scheme 23).

**Scheme 24:**
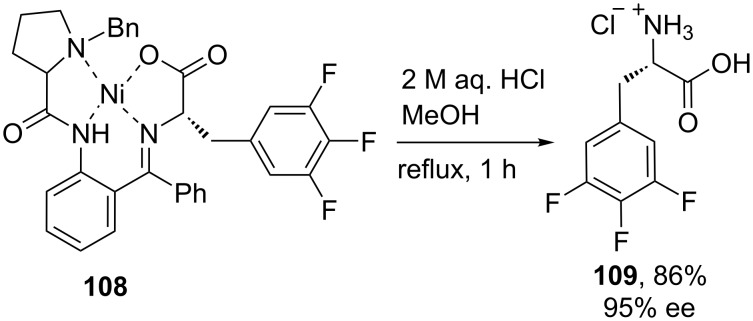
Synthesis of 3,4,5-trifluorophenylalanine hydrochloride (**109**).

Following the previous method, the chiral auxiliary **108** was readily cleaved under mild acidic conditions to afford the hydrochloride salt of 3,4,5-trifluoro-Phe **109** in 86% yield and 95% ee, indicating very low racemization [62] (Scheme 24).

#### 1.10. PAM enzymatic catalytic amination of (*E*)-cinnamic acid

**Scheme 25:**
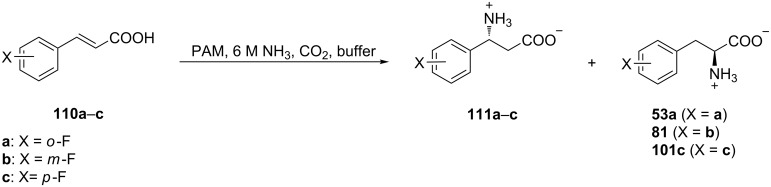
Synthesis of FPhe derivatives via phenylalanine aminomutase (PAM).

The enzyme phenylalanine aminomutase (PAM) from *Taxus chinensis* catalyzes the stereoselective isomerization of α-phenylalanine to β-phenyalanine **111a–c**. Mechanistic studies showed that (*E*)-cinnamic acid is an intermediate in this transformation [63]. Accordingly, addition of ammonia to *o*, *m*, or *p-*fluoro-(*E*)-cinnamic acids **110a–c** catalyzed by PAM afforded (*R*)-fluoro-β-phenylalanines **111a–c** and *o*, *m*, and *p*-(*S*)-fluorophenylalanines **53a**, **81**, **101c**, respectively, with excellent enantioselectivities (**<**99% ee) (Scheme 25).

#### 1.11. From enamine intermediates

**Scheme 26:**
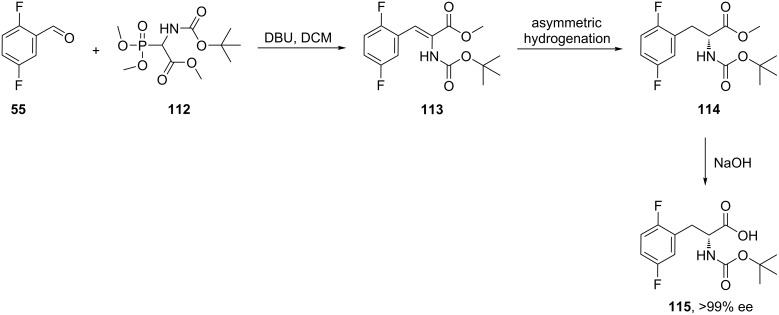
Synthesis of (*R*)-2,5-difluorophenylalanine **115**.

The synthesis (*R*)-2,5-difluorophenylalanine derivative **115** was carried out by coupling the commercially available aldehyde **55** and *N*-Boc phosphonate glycinate **112** to generate the enamino ester intermediate **113**. The asymmetric hydrogenation of this enamine afforded the *N*-Boc-protected (*R*)-2,5-difluorophenylalanine ester **114** with >99% ee. A following alkaline hydrolysis of the ester **114** gave *N*-Boc-(*R*)-2,5-difluorophenylalanine **115** (Scheme 26) [50].

After compiling the above synthetic methods, a number of conclusions can be drawn regarding the synthesis of FPhe analogues of type **I** and **II**. The most convenient method involved a Negishi cross coupling of an aryl halide and the Zn homoenolate of the protected (*R*)-iodoalanine **2** using a Pd(0) catalyst. This method provided a versatile range of fluorinated phenylalanine products with high enantioselectivities and in acceptable yields.

### 2. Synthesis of β-fluorophenylalanines of type **III**

#### 2.1. Fluorination of protected (1*R*,2*R*)-2-amino-ʟ-phenylpropane-1,3-diol

**Scheme 27:**
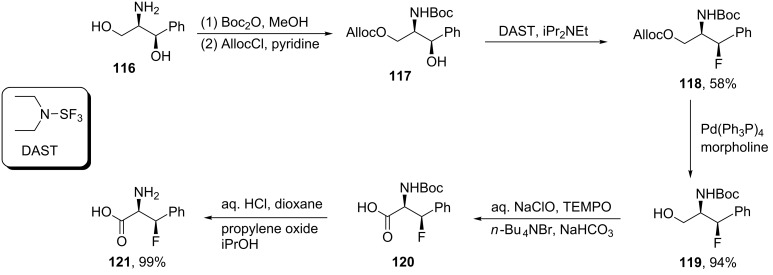
Synthesis of β-fluorophenylalanine via 2-amino-1,3-diol derivatives.

Recently, Okuda et al. reported the synthesis of (3*R*)-3-fluoro-ʟ-phenylalanine (**121**) from (1*R*,2*R*)-2-amino-ʟ-phenylpropane-1,3-diol (**116**). Thus, Boc-protection of the amine group in **116** followed by the protection of the primary hydroxy group (Alloc) gave alcohol **117** in good yield. The fluorination of **117** was achieved by treatment with DAST to form **118**. Then, selective removal of the Alloc protecting group using Pd(PPh_3_)_4_, was followed by oxidation of the resulting Boc-protected amino alcohol **119** to give the *N*-Boc-protected acid **120** in good yield. Finally, removal of the Boc group then generated the free amino acid (3*R*)-3-fluoro-ʟ-phenylalanine (**121**) [64] (Scheme 27). Okuda’s group used **121** in the synthesis of a nucleoside that could be used to assess the transition state of a ribosome-catalyzed peptide-bond formation [64].

#### 2.2. Stereoselective benzylic fluorination of *N*-(2-phenylacetyl)oxazolidin-2-one using NFSI

**Scheme 28:**
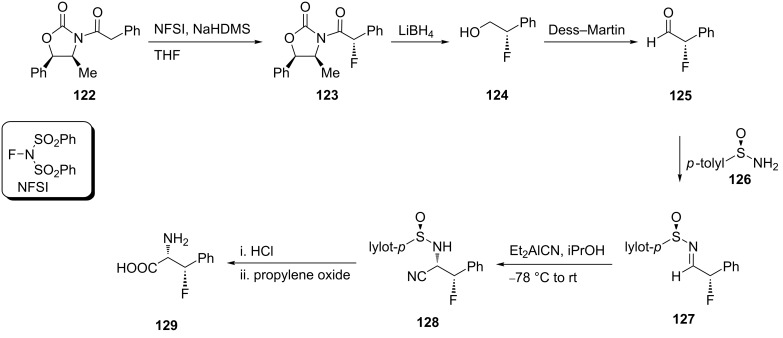
Synthesis of β-fluorophenylalanine derivatives via the oxazolidinone chiral auxiliary **122**.

Treatment of oxazolidinone **122** with *N*-fluorobenzenesulfonimide (NFSI) in the presence of NaHMDS afforded the fluorinated oxazolidinone derivative **123**. The reductive removal of the chiral auxiliary with LiBH_4_ resulted in alcohol **124** which was oxidized by Dess–Martin periodinane to give (*S*)-(−)-2-fluoro-2-phenylacetaldehyde (**125**). This aldehyde is prone to racemization and decomposition and therefore was directly converted to the arylidene derivative **127**, by treatment with *p*-toluenesulfinamide (**126**). Then, reaction of **127** (1.0 mmol) with 1.5/1.0 equiv of diethylaluminum cyanide (Et_2_AlCN)/iPrOH at −78 °C in THF gave nitrile **128**. Deprotection of the latter, followed by hydrolysis of the nitrile group afforded *syn*-(2*S*,3*S*)-(+)-3-fluorophenylalanine (**129**) [65] (Scheme 28) .

#### 2.3. Multistep synthesis from ethyl trifluoropyruvate hemiketal

**Scheme 29:**
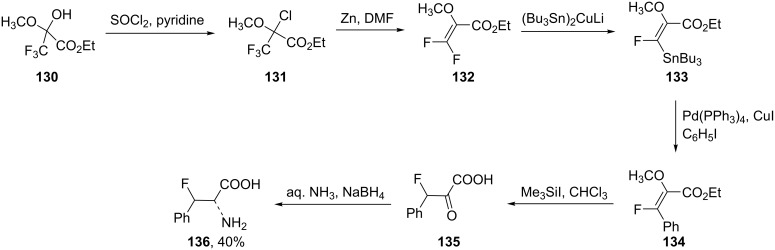
Synthesis of β-fluorophenylalanine from pyruvate hemiketal **130**.

The reaction of ethyl trifluoropyruvate hemiketal **130** with thionyl chloride in pyridine afforded the chlorinated derivative **131**, which upon treatment with zinc powder in DMF, afforded the dihalogenated olefin **132**. The substitution of one fluorine atom in **132** with a tributylstannyl group to give **133** was accomplished by the reaction with (Bu_3_Sn)_2_CuLi in THF at −78 °C. The reaction took place following an addition–elimination mechanism. Then, coupling of **133** with iodobenzene in the presence of Pd(PPh_3_)_4_ and CuI as the co-catalyst afforded ethyl (*E*)-3-phenyl-3-fluoro-2-methoxypropenoate (**134**) which was converted into the corresponding α-ketoacid **135** by treatment with trimethylsilyl iodide. Finally, the reaction of **135** with aqueous ammonia followed by reduction with sodium borohydride gave the racemic *erythro*-β-fluorophenylalanine **136** in 40% yield [66] (Scheme 29).

#### 2.4. Fluorodehydroxylation of β-hydroxyphenylalanine

**Scheme 30:**
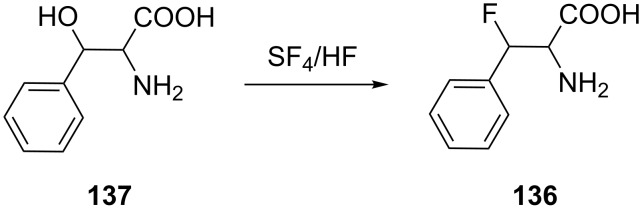
Synthesis of β-fluorophenylalanine (**136**) via fluorination of β-hydroxyphenylalanine (**137**).

Alternatively, Kollonitach et al. prepared racemic 3-fluorophenylalanine (**136**) through the fluorodehydroxylation of 3-hydroxyphenylalanine (**137**) using sulfur tetrafluoride (SF_4_) in HF [67] (Scheme 30).

#### 2.5. Ring opening of aziridine derivatives by HF/Py

**Scheme 31:**
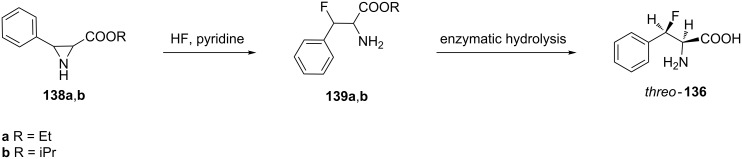
Synthesis of β-fluorophenylalanine from aziridine derivatives.

The ring opening reaction of aziridines **138a**,**b** by treatment with hydrogen fluoride in pyridine afforded 3-fluorophenylalanine esters **139a,b**. The subsequent enzymatic hydrolysis of esters **139a**,**b** gave the *threo*-isomer **136** in an enantiomerically pure form [68–69] (Scheme 31).

On the other hand, Wade et al. reported that ester **138b** (R = iPr) afforded the isopropyl 3-fluorophenylalaninate (**139b**) as racemate in 45–50% yield [70] under similar reaction conditions (Scheme 31).

#### 2.6. Fluorination and reductive amination of phenylpyruvate

**Scheme 32:**
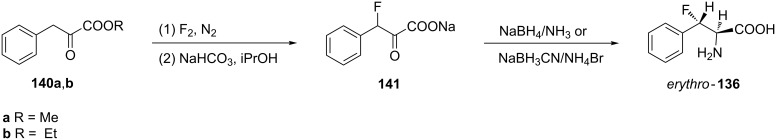
Synthesis of β-fluorophenylalanine **136** via direct fluorination of pyruvate esters.

A direct fluorination of the ester derivatives of phenylpyruvic acids **140a**,**b** with F_2_ followed by hydrolysis of the resulting fluoropyruvates in 50% isopropanol in the presence of NaHCO_3_ gave 3-fluoro-3-phenylpyruvate **141** in 40–50% yields [68]. The direct reductive amination gave a partially racemized mixture of *threo* and *erythro*-**136** with the *erythro* stereoisomer **136** as the major product **(**Scheme 32).

**Scheme 33:**
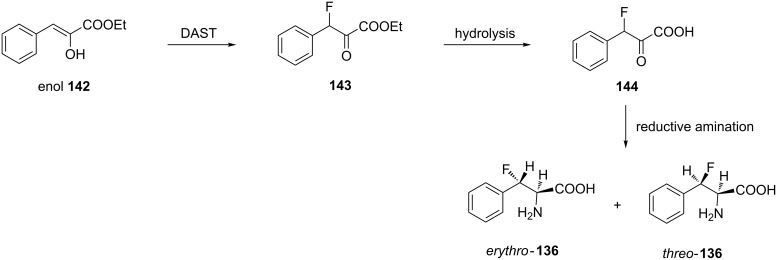
Synthesis of β-fluorophenylalanine via fluorination of ethyl 3-phenylpyruvate enol using DAST.

The reductive amination of 3-fluoro-3-phenylpyruvic acid (**144**) obtained by the fluorodehydroxylation of the enol form of ethyl 3-phenylpyruvate **142**, using DAST instead of SF_4_ followed by hydrolysis, produced both *threo* and *erythro-*diastereomers of **136** [68] (Scheme 33).

#### 2.7. Photocatalyzed benzylic fluorination of *N*-phthalimido phenylalanine

**Scheme 34:**
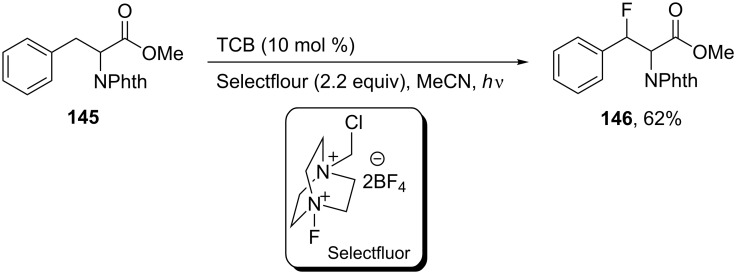
Synthesis of β-fluorophenylalanine derivatives using photosensitizer TCB.

The photocatalyzed benzylic fluorination of phthalimide-protected phenylalanine methyl ester **145**, using the photosensitizer 1,2,4,5-tetracyanobenzene (TCB), and Selectfluor in acetonitrile was carried out using a pen lamp (λ_max_ = 302 nm). By this route, the β-fluoro derivative **146** was obtained in 62% yield as racemic mixture [71] (Scheme 34). Recently, Egami and coworker also synthesized compound **146** in 43% yield (dr = 1:1) via the fluorination of **145**, however without TCB as photosensitizer, but instead using an LED light source (365 nm) and Selectfluor in MeCN [72].

**Scheme 35:**
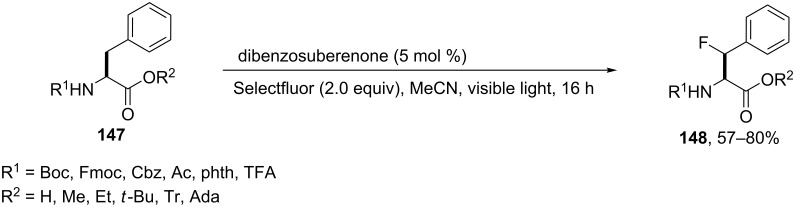
Synthesis of β-fluorophenylalanine derivatives using Selectflour and dibenzosuberenone.

Alternatively, a visible light (14 Watt CFL) mediated benzylic fluorination of a series of *N*- and *C*-terminally protected phenylalanines **147** using Selectfluor and dibenzosuberenone in acetonitrile, afforded the β-fluorophenylalanine derivatives **148** in variable yields with partial racemization. Phthalimido and trifluoroacetyl *N*-terminal protecting groups (R^1^ = Phth or TFA) and unprotected *C*-terminal derivatives (R^2^ = H) provided the most efficient outcomes (80 and 67% yield, respectively). An *N*-acetyl group was also suitable as protecting group for the reaction providing the desired product with 57% yield. Also, methyl and ethyl esters as *C*-terminal protecting groups in combination with phthalimino as the *N*-terminal protecting group, were both successfully explored. However, when the trifluoroacetyl amide was used as a substrate the methyl ester performed better than the ethyl ester (74% versus 60% yield). However, *N-*protecting groups such as Boc, Fmoc, and Cbz were not compatible with the fluorination (0–10% yield). Moreover, when *tert*-butyl, trityl, and adamantyl protecting groups were installed for *C*-terminal protection additional fluorination, decomposition, and consequently low yields of the β-fluorinated derivatives **148** were observed [73] (Scheme 35).

#### 2.8. Fluorination of aziridinium derivatives

**Scheme 36:**
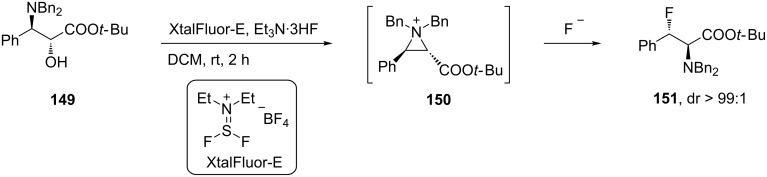
Synthesis of protected β-fluorophenylalanine via aziridinium intermediate **150**.

The *N*,*N*-dibenzylated 3-fluorophenylalanine derivative **151** was prepared with excellent diastereoisomeric ratio (dr > 99:1) from α-hydroxy-β-amino ester **142**. In this case, XtalFluor-E was used to activate the OH group in the substrate and displaced by neighboring amino-group participation creating an aziridinium intermediate **150**. The latter then was opened stereo- and regioselectively by fluoride to give **151** in good yield and high diastereoisomeric purity (Scheme 36). The subsequent deprotection of **151** had to be achieved with BrO_3_^−^, because hydrogenolysis resulted in defluorination [74].

**Scheme 37:**
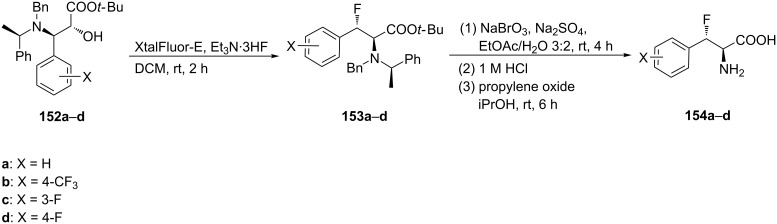
Synthesis of β-fluorophenylalanine derivatives via fluorination of α-hydroxy-β-aminophenylalanine derivatives **152**.

Alternatively, a series of substituted *anti*-β-fluorophenylalanine derivatives **154a**–**d** was obtained from the corresponding enantiopure α‑hydroxy-β-aminophenylalanine esters [75–76] **152a**–**d** using XtalFluor-E. The reaction also included an aziridinium ion rearrangement as the key step. Deprotection of the resultant β-fluoro-α-amino acid esters **153a**–**d** afforded the corresponding enantiopure *anti*-β-fluorophenylalanines **154a**–**d** in good yield and high diastereoisomeric purities [74] (Scheme 37).

**Scheme 38:**
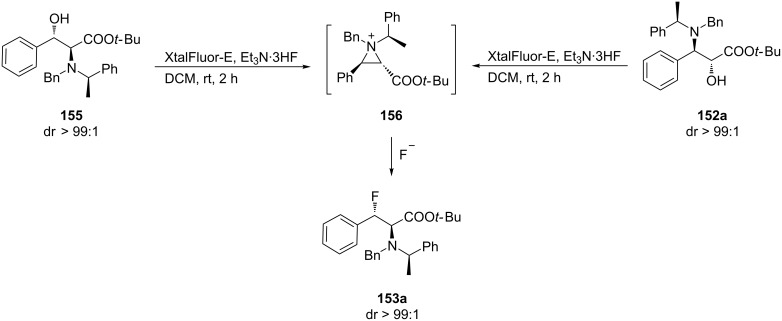
Synthesis of β-fluorophenylalanine derivatives from α- or β-hydroxy esters **152a** and **155**.

The deoxyfluorination of the enantiopure α-hydroxy-β-amino ester **152a** or α-amino-β-hydroxyphenylalanine ester **155** [74–76] under the same conditions proceeded via aziridinium ion **156** and generated β-fluoro-α-amino ester **153a** in good yield and high diastereoisomeric purity (dr > 99:1) (Scheme 38).

#### 2.9. Direct fluorination of β-methylene C(sp^3^)−H

**Scheme 39:**
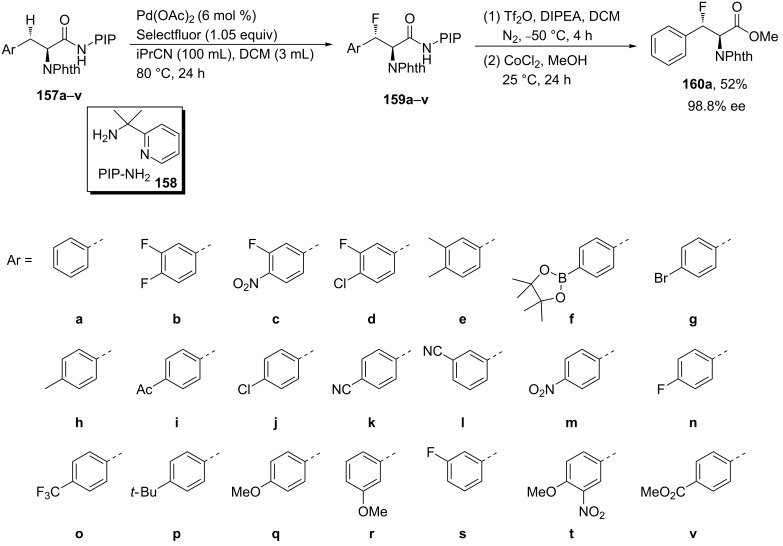
Synthesis of a series of β-fluoro-Phe derivatives via Pd-catalyzed direct fluorination of β-methylene C(sp^3^)–H bonds in Phe substrates functionalized with the PIP auxiliary group.

The direct fluorination of β-methylene C(sp^3^)−H bonds of Phe derivatives **157a–v** having installed the bidentate auxiliary, 2-(pyridine-2-yl)isopropylamine (PIP-amine) **158**, was attempted using Selectfluor in the presence of Pd(OAc)_2_ as a catalyst. The corresponding β-fluoro derivatives **159a–v** were obtained with high stereoselectivity using DCM and iPrCN as co-solvent (30:1 v/v). Interestingly reducing the Pd(OAc)_2_ concentration from 10 mol % to 6 mol % led to an improved yield of 73% for **159a**, **159g**, and **159t**. Selectfluor was found superior to other fluorination reagents such as fluoropyridinium tetrafluoroborate, 2,4,6-trimethylfluoropyridinium tetrafluoroborate, or NFSI. The fluorination process was explored with a broad range of substituted Phe derivatives. The removal of the PIP auxiliary group without affecting the newly introduced fluorine atom was attempted by a two-step, one-pot protocol involving an in situ esterification of a highly electrophilic pyridinium triflate intermediate [77] and afforded the *anti*-β-fluoro-α-amino acid methyl ester **160a** in 52% yield and with 98.8% ee (Scheme 39).

**Scheme 40:**
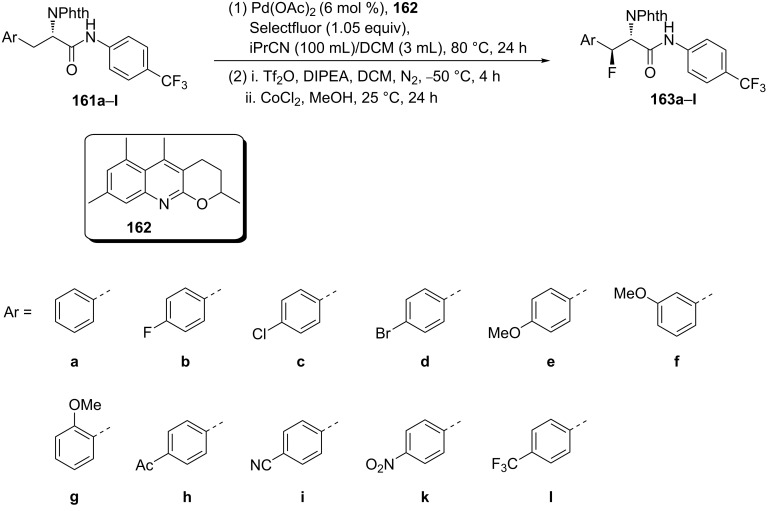
Synthesis of series of β-fluorinated Phe derivatives using quinoline-based ligand **162** in the Pd-catalyzed direct fluorination of β-methylene C(sp^3^)–H bonds.

On the other hand, when the quinoline-based ligand **162** was used, it was shown to promote the palladium-catalyzed direct electrophilic fluorination of β-methylene C(sp^3^)–H bonds. Thus, fluorinations of ʟ-phenylalanine 4-trifluoromethylphenylamides **161a–l** carrying a range of functional groups such as fluoro, chloro, bromo, methoxy, acetyl, cyano, nitro, and trifluoromethyl, were well-tolerated and afforded the corresponding *anti*-β-fluoro-α-amino acids **163a–l** in moderate to excellent yields [78] (Scheme 40).

### 3. Synthesis of β,β-difluorophenylalanine derivatives of type **IV** via 2-phenyl-2,2-difluoroacetaldehyde derivatives

**Scheme 41:**
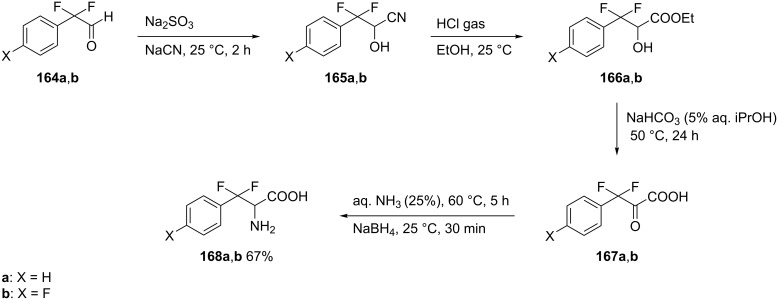
Synthesis of β,β-difluorophenylalanine derivatives from 2,2-difluoroacetaldehyde derivatives **164a**,**b**.

2-Phenyl- and 2-(4-fluorophenyl)-2,2-difluoroacetaldehyde **164a** and **164b** proved to be key starting points for the synthesis of β,β-difluorophenylalanine analogs **168a**,**b**. The conversion of **164a**,**b** into their respective cyanohydrins **165a**,**b** followed by acid hydrolysis with gaseous HCl in ethanol afforded the α-hydroxy esters **166a**,**b**. Dess–Martin oxidation [79–80] of the latter, followed by hydrolysis of the ester gave keto acids **167a**,**b**. Finally, the reductive amination of **167a**,**b** with 25% aqueous ammonia and NaBH_4_ afforded the racemic β,β-difluorophenylalanine derivatives **168a**,**b** in 67% yield [81] (Scheme 41).

**Scheme 42:**
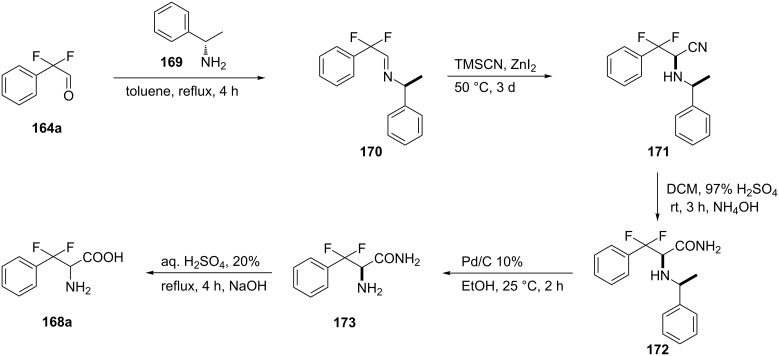
Synthesis of β,β-difluorophenylalanine derivatives via an imine chiral auxiliary.

An alternative approach to the difluorinated compound **168a** was achieved by the condensation of **164a** with (*S*)-1-phenylethylamine (**169**), to give the imine **170**. Heating of imine **170** with TMSCN in the presence of zinc iodide [82] generated the nitrile **171** as a 1:1 mixture of diastereoisomers which was immediately hydrolyzed to provide the racemic carboxamide **172**. The subsequent removal of the chiral auxiliary by catalytic hydrogenation then afforded the carboxamide **173**. Finally, an acid-mediated hydrolysis of the carboxamide **173** to generate the free amino acids ʟ- or ᴅ-**168a**, was carried out with aqueous H_2_SO_4_. However, the acid hydrolysis step was accompanied with extensive racemization [81,83] (Scheme 42).

### 4. Synthesis of α-fluorophenylalanine of type **V** via α-fluorination of Phe derivatives

**Scheme 43:**
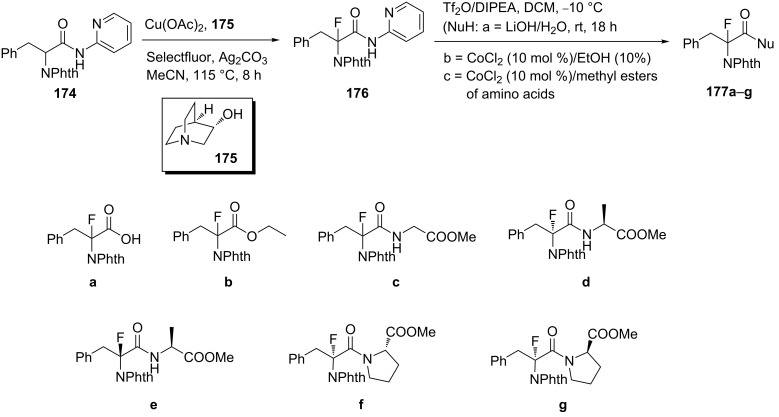
Synthesis of α-fluorophenylalanine derivatives via direct fluorination of protected Phe **174**.

The successful α-fluorination of phenylalanine derivative **174** carrying a picolinamide auxiliary to give **176** was carried out using Selectfluor as the fluorination reagent. The direct α-C(sp^3^)–H fluorination of the starting compound **174** was catalyzed by Cu(OAc)_2_ with (*R*)-3-hydroxyquinuclidine ligand **175**. Comparative studies demonstrated that using ligand **175** rather than other ligands gave higher yields and no β*-*elimination products. The effective removal of the auxiliary using triflic anhydride with LiOH as nucleophile, gave product **177a** in good yield. Alternative nucleophiles such as EtOH or methyl esters of amino acids, in the presence of catalytic amounts of CoCl_2_, afforded product **177b** or fluorinated Phe dipeptides [84] **177c–g** as racemic mixtures (Scheme 43).

### 5. Pharmaceutical applications of fluorinated phenylalanine derivatives

Peptides and proteins containing FPhe are important tools to identify enzyme–substrate complexes, mechanisms of protein aggregation, and modifying the chemical and thermal stabilities of proteins. The properties of protein were preserved, when low levels of fluorine are incorporated into the constituent amino acids, and were comparable with that of the original proteins. Helpfully, fluorine incorporation may favorably adjust protein function including improved stability and substrate selectivity.

#### 5.1. Applications of FPhe derivatives in positron emission tomography (PET)

The molecular imaging technique positron emission tomography (PET) provides information on tumor metabolism, which allows for a more accurate diagnostic and therapy response in neuro-oncology, compared to, for example, magnetic resonance imaging (MRI). PET is particularly well-suited to differentiate neoplastic tissue from non-specific changes induced by chemotherapy treatments [85]. PET is particularly used for the early detection of tumors and metastases, and is an established tool for the diagnosis, staging, and the treatment planning of various malignancies. The selective imaging of tumors using PET exploits radiotracers that target aberrant cellular metabolism or increased protein expression [86–87]. Here, the ^18^F isotope is particularly useful for the preparation of radiotracers to be used in PET due to its relatively long half-life (109 min). In this section we highlight two selected ^18^FPhe derivatives which are used for PET tumor detection. 4-Borono-2-[^18^F]fluoro-ᴅ,ʟ-phenylalanine ([^18^F]FBPA, **70**), is a fluorinated derivative of the parent compound designed for boron neutron capture therapy (BNCT) [53,88–92]. This compound was used for PET imaging of melanoma in animal models.

**Figure 2:**
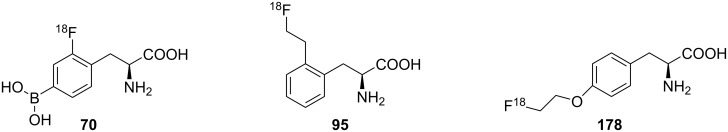
Structures of PET radiotracers of ^18^FPhe derivatives.

The low affinity of **178** for the ʟ-type amino acid transporter1 (LAT1), however, limited the use of this compound as PET radiotracer for brain tumor imaging [93–96]. Therefore, for further analysis and comparison with **178** (performed in vitro), as the most promising candidate 2-[^18^F]-2-fluoroethyl-ʟ-phenylalanine (2-[^18^F]FELP, **95**) was selected. In a F98 glioblastoma rat model, 2-[^18^F]FELP exhibited improved in vitro characteristics over [^18^F]FET **178**, especially in view of the affinity and specificity for system L [58]. Accordingly, 2-[^18^F]FELP **95** emerged as a promising PET radiotracer for brain tumor imaging [97–101] (Figure 2).

#### 5.2. Incorporation of FPhe for the synthesis of fluorinated drugs

**Figure 3:**
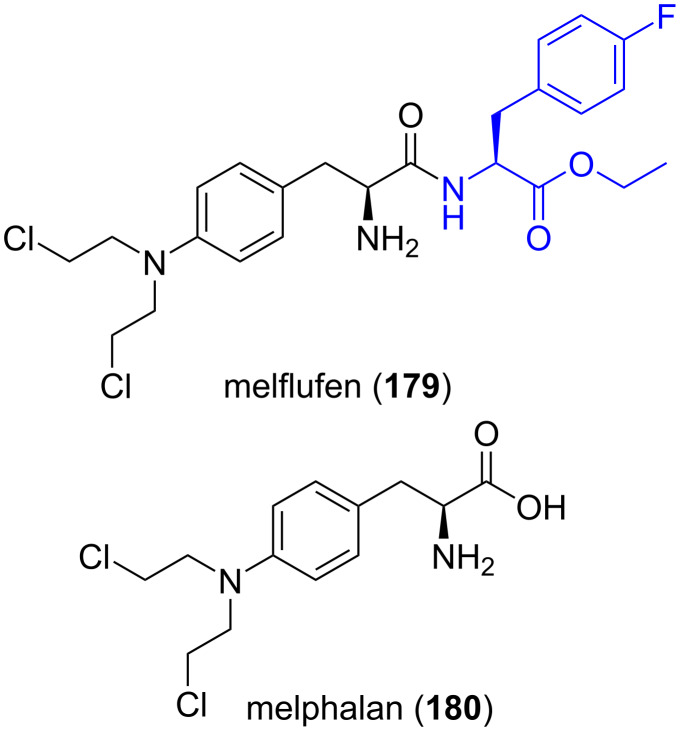
Structures of melfufen (**179**) and melphalan (**180**) anticancer drugs.

**5.2.1. Melflufen, an anticancer drug:** 4-Fluoro-ʟ-phenylalanine ester is required for the synthesis of melflufen (**179**), an anticancer drug currently being in clinical trials for the treatment of relapsed and refractory multiple myeloma (RRMM) [102–103]. Melflufen is a next generation form of the more historical drug, melphalan **180** (Figure 3).

**Figure 4:**
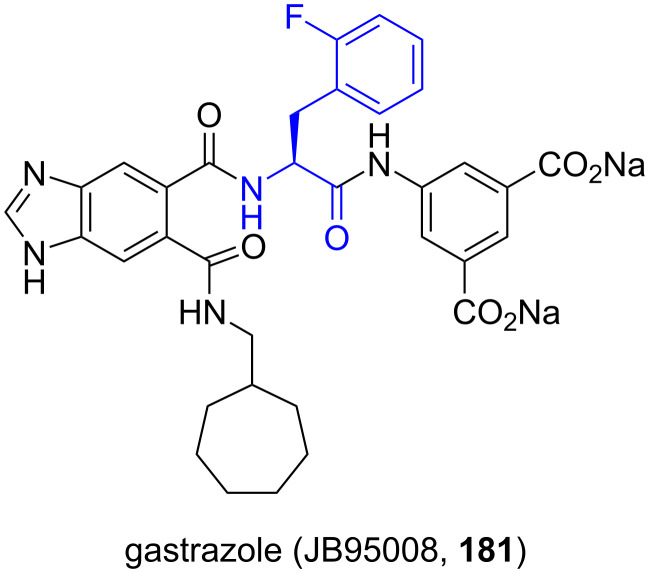
Structure of gastrazole (JB95008, **181**), a CCK2 receptor antagonist.

**5.2.2. Gastrazole (JB95008), a CCK2 receptor antagonist:** 2-Fluoro-ʟ-phenylalanine derivatives are required for the synthesis of gastrazole (JB95008, **181**), a potent and highly selective cholecystokinin-2 (CCK2) receptor antagonist, originally developed at the James Black Foundation [104–109]. Roberts et al. demonstrated its inhibitory activity of gastrin-stimulated growth of pancreatic cancer both in vitro and in vivo studies [110] (Figure 4).

**Figure 5:**
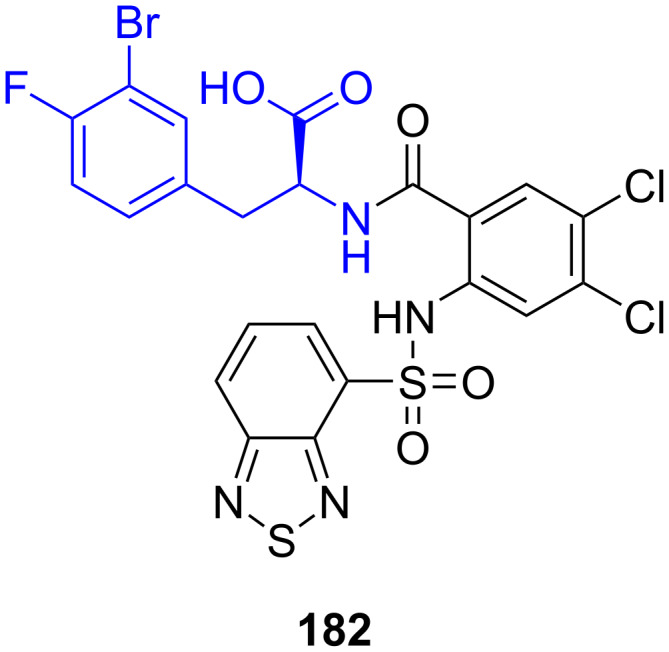
Dual CCK1/CCK2 antagonist **182**.

**5.2.3. Dual CCK1/CCK2 receptor antagonists:** Johnson & Johnson identified compound **182** as a dual CCK1/CCK2 receptor antagonist with desirable pharmacologic properties [51,111–113] (Figure 5). As can be seen from the structure of **182**, 3-bromo-4-fluoro-ʟ-phenylalanine (**65**) is required for the synthesis.

**Figure 6:**
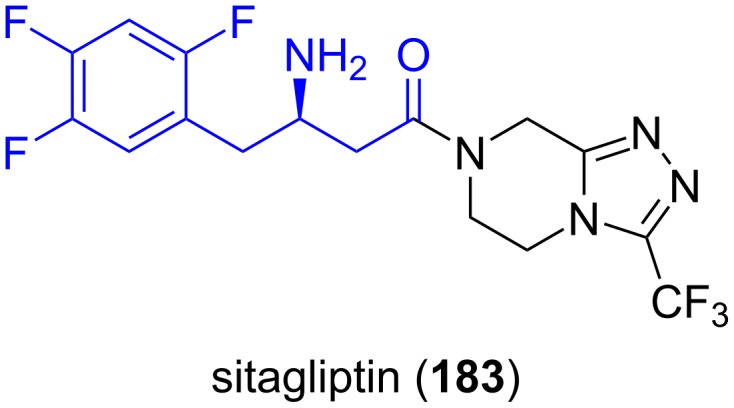
Structure of sitagliptin (**183**), an antidiabetic drug.

**5.2.4. Antidiabetes drugs, sitagliptin:** (*R*)-2,4,5-Trifluorophenylalanine **38b** is a constituent of sitagliptin (**183**, Figure 6). Sitagliptin is used to decrease the level of blood sugar in patients with type 2 diabetes and belongs to the dipeptidyl peptidase-4 (DPP-4) class of inhibitors [114–115]. This enzyme breaks down the incretins GLP-1 and GIP, gastrointestinal hormones released in response to a meal. By preventing the breakdown of GLP-1 and GIP, they are able to increase the secretion of insulin by the pancreas that modulates blood sugar level when it is high. Sitagliptin was granted FDA approval in October, 2006 [116].

**Figure 7:**
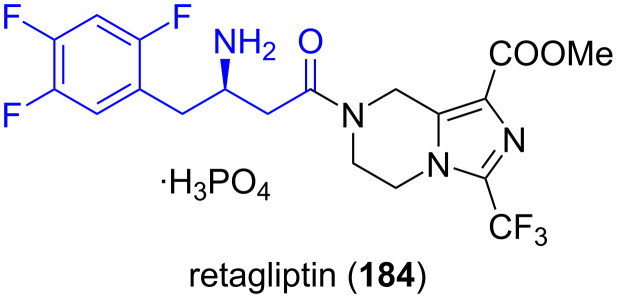
Structure of retaglpitin (**184**) and antidiabetic drug.

**Retagliptin phosphate:** Retagliptin phosphate (**184**) is under investigation as a DPP-4 inhibitor for treating type-2 diabetes. It is an analogue of sitagliptin which was developed for the same application [109,117], but compound **184** appears to have an improved activity [118]. Retagliptin showed efficacy in clinical trials and is now entering phase III studies. (*R*)-2,4,5-Trifluorophenylalanine **38b** is used as a building block in the synthesis of compound **184** [119–120] (Figure 7).

**Figure 8:**
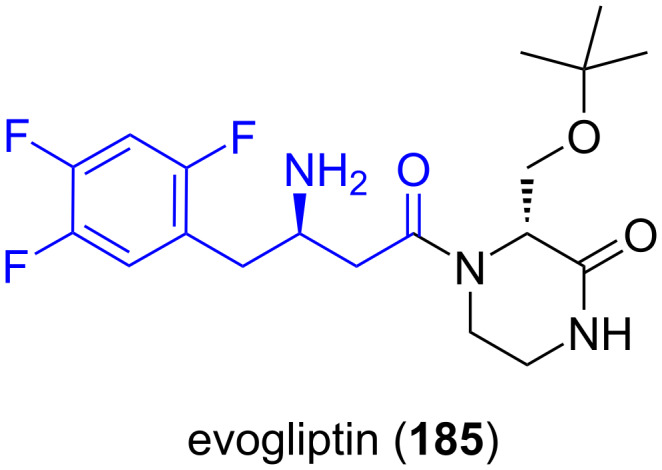
Structure of evogliptin (**185**), an antidiabetic drug.

**Evogliptin:** (*R*)-2,4,5-Trifluorophenylalanine **38b** is required for the synthesis of evogliptin (**185**, Figure 8), an antidiabetic drug in the dipeptidyl peptidase-4 (DPP-4) inhibitor or "gliptin" class of drugs. The South Korean pharmaceutical company Dong-A ST developed evogliptin (**185**) and it is currently approved for use in South Korea [121–123].

**Figure 9:**
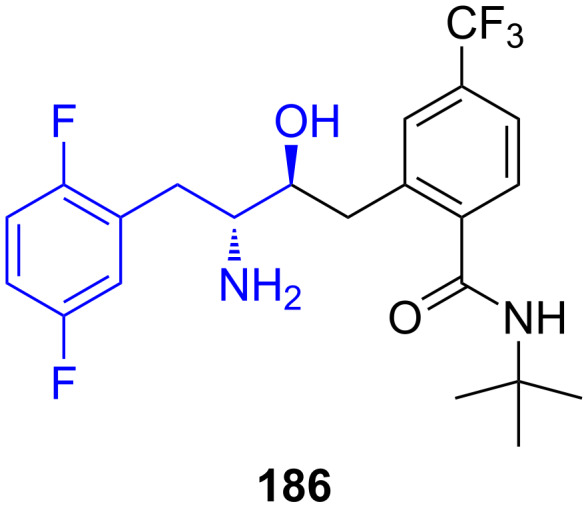
Structure of LY2497282 **(186**) a DPP-4 inhibitor for the treatment of type II diabetes.

**LY2497282:** Eli Lilly identified LY2497282 (**186**) as a potent and selective DPP-4 inhibitor, also for the treatment of type II diabetes. The inhibition of GLP-1 degradation by dipeptidyl peptidase IV (DPP-4) has emerged as a promising approach for treatment. (*R*)-2,5-Difluorophenylalanine is a required building block for the synthesis of LY2497282 [50,124] (Figure 9).

**Figure 10:**
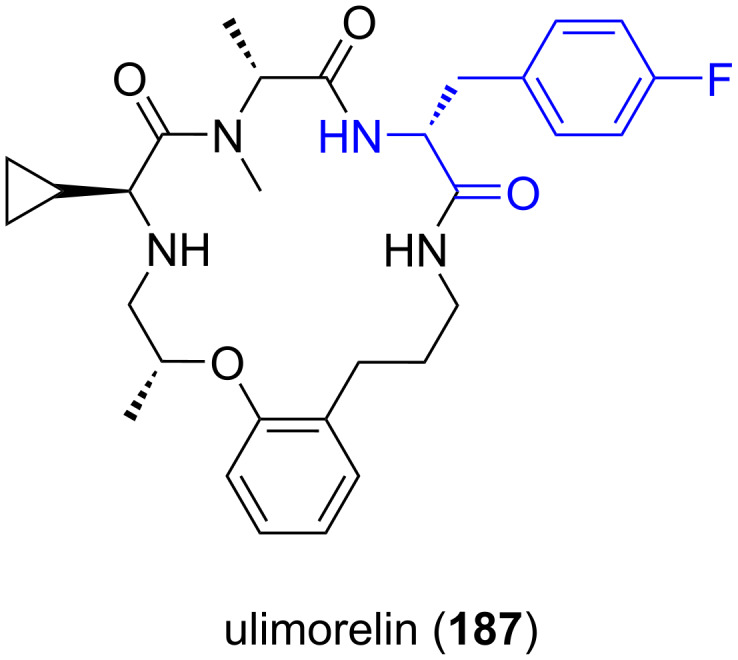
Structure of ulimorelin (**187**).

**5.2.5. Ulimorelin:** Ulimorelin (**187**) is a small cyclic peptide containing ᴅ-4-FPhe. Ulimorelin acts as a selective agonist of the ghrelin/growth hormone secretagogue receptor (GHSR-1A), which is currently being developed by Tranzyme Pharma (code name TZP-101) as a first-in-class treatment for both, postoperative ileus (POI) and diabetic gastroparesis. POI describes a deceleration or arrest in intestinal motility following surgery [109,125–127] (Figure 10).

**Figure 11:**
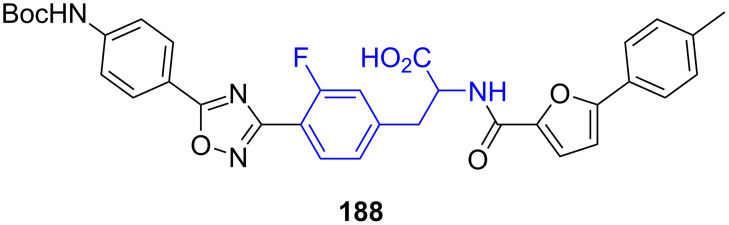
Structure of GLP1R (**188**).

**5.2.6. The glucagon-like peptide-1 receptor (GLP1R):** 3’-Fluorophenylalanine is a key motif in the structure of the glucagon-like peptide-1 receptor (GLP1R, **188**, Figure 11). GLP1R is a receptor protein found on beta cells of the pancreas and on neurons of the brain. It is participating in the modulation of blood sugar levels by increasing insulin secretion. Consequently, GLP1R plays a key role in the development of drugs to treat diabetes mellitus [128–130].

**Figure 12:**
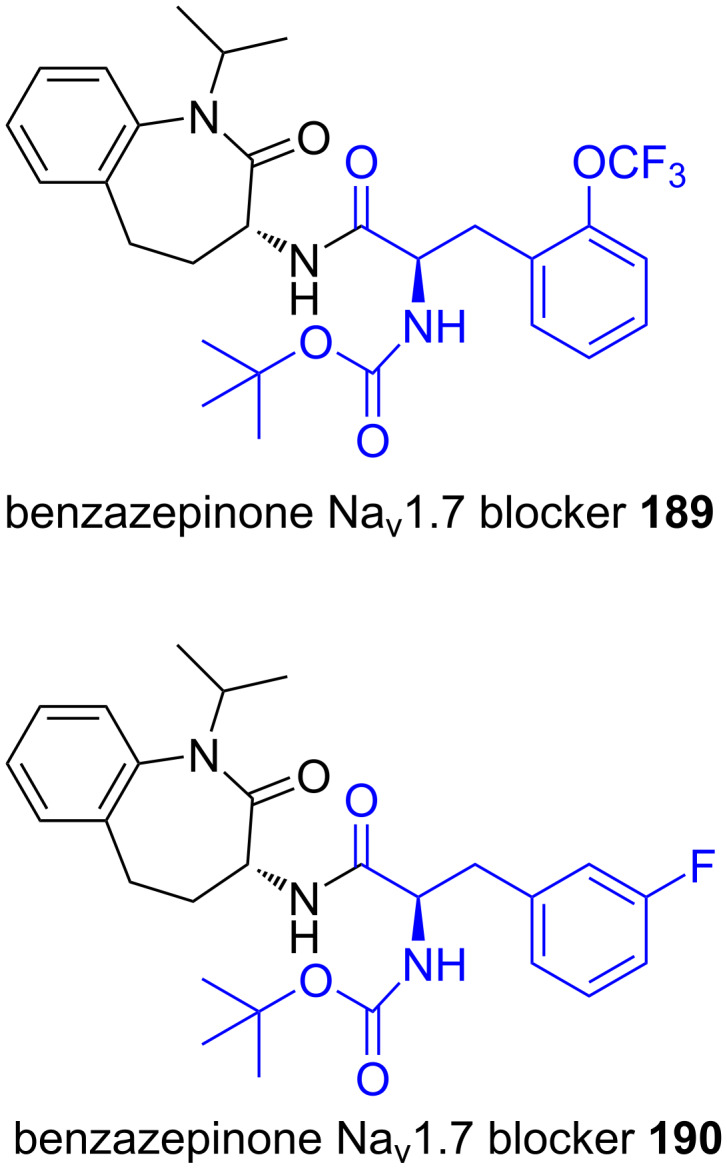
Structures of Nav1.7 blockers **189** and **190**.

**5.2.7. Sodium channel blockers (benzazepinone Nav1.7 blocker):** Sodium channel blockers are used in the treatment of neuropathic pain. This is a chronic, debilitating condition that results from injury of the peripheral or central nervous system and can be triggered by a variety of events or conditions, including diabetes, shingles and chemotherapy [131]. Merck reported [132] the discovery of a structurally novel class of benzazepinone hNav1.7 voltage-gated sodium channel blockers containing 2-trifluoromethoxy-ʟ-phenylalanine derivative **189** and 3-ʟ-FPhe **190** (Figure 12) [133]. Compounds **189** and **190** were investigated as potential drugs for the treatment of neuropathic pain because they inhibited action potential firing. It was suggested, based on genetic studies, that a selective Nav1.7 inhibition, will produce robust inhibition of pain without significant side effects [134–135].

## Conclusion

In view of the increased significance of FAAs in the development of bioactive compounds, considerable efforts were dedicated to the development of efficient synthetic protocols to FAAs. Among them, a range of fluorinated phenylalanines emerged, that have enhanced the biophysical, chemical and biological properties of bioactives. Accordingly, synthetic approaches to five distinct classes of fluorinated analogues were reviewed here. Synthetic protocols and strategies varied according to the position of the fluorine substituent. Also included were ^18^FPhe derivatives, some of which emerged as promising radiotracers in positron emission tomography (PET). Finally, it is notable that there are a significant number of FPhe derivatives which are nowadays incorporated into drug scaffolds of compounds either licensed or currently being studied in clinical trials.

**Table 1:** List of abbreviations.

Ada	adamantyl
Alloc	alloxycarbonyl
BNCT	boron neutron capture therapy
Boc	*tert*-butoxycarbonyl
Cbz	benzyloxycarbonyl
CCK	cholecystokinin
DAST	diethylaminosulfur trifluoride
DIBAL	diisobutylaluminium hydride
DMB	dimethoxybenzyl
DMAc	dimethylacetamide
DPP-4	dipeptidyl peptidase-4
DMPU	*N,N*′-dimethylpropyleneurea
FAAs	fluorinated amino acids
FPhe	fluorinated phenylalanine
Fmoc-Osu	*N*-(9-fluorenylmethoxycarbonyloxy)succinimide
GLP1R	glucagon-like peptide-1 receptor
kryptofix^®^222	4,7,13,16,21,24-hexaoxa-1,10-diazabicyclo[8.8.8]hexacosane
LAT1	L-type amino acid transporter1
LHMDS	lithium bis(trimethylsilyl)amide
LDA	lithium diisopropylamide
NaHDMS	sodium bis(trimethylsilyl)amide
NFSI	*N*-fluorobenzenesulfonimide
PAM	phenylalanine aminomutase
PET	positron emission tomography
PIP	2-(pyridin-2-yl)isopropylamine
Phth	phthalimido
RRMM	relapsed and refractory multiple myeloma
Selectfluor	1-chloromethyl-4-fluoro-1,4-diazoniabicyclo[2.2.2]octane bis(tetrafluoroborate)
SPhos	2-dicyclohexylphosphino-2′,6′-dimethoxybiphenyl
TBAF	tetrabutylammonium fluoride
TMSI	trimethylsilyl iodide
TMSCN	trimethylsilyl cyanide
TCE	trichloroethyl
TCB	1,2,4,5-tetracyanobenzene
Tr	triphenylmethyl
TEMPO	2,2,6,6-tetramethylpiperidin-1-yl)oxyl or (2,2,6,6-tetramethylpiperidin-1-yl)oxidanyl
TBTA	*tert-*butyl-2,2,2-trichloroacetimidate
TCNHPI	*N*-hydroxytetrachlorophthalimide
XtalFluor-E	*N,N*-diethylamino-*S*,*S*-difluorosulfinium tetrafluoroborate

## References

[1] Remete A M, Nonn M, Fustero S, Fülöp F, Kiss L (2018). Tetrahedron.

[2] Salwiczek M, Nyakatura E K, Gerling U I M, Ye S, Koksch B (2012). Chem Soc Rev.

[3] Merkel L, Budisa N (2012). Org Biomol Chem.

[4] Purser S, Moore P R, Swallow S, Gouverneur V (2008). Chem Soc Rev.

[5] Odar C, Winkler M, Wiltschi B (2015). Biotechnol J.

[6] Gómez-Nuñez M, Haro K J, Dao T, Chau D, Won A, Escobar-Alvarez S, Zakhaleva V, Korontsvit T, Gin D Y, Scheinberg D A (2008). PLoS One.

[7] Noren C, Anthony-Cahill S, Griffith M, Schultz P (1989). Science.

[8] Ye S, Berger A A, Petzold D, Reimann O, Matt B, Koksch B (2010). Beilstein J Org Chem.

[9] Tang Y, Ghirlanda G, Petka W A, Nakajima T, DeGrado W F, Tirrell D A (2001). Angew Chem, Int Ed.

[10] Bilgiçer B, Fichera A, Kumar K (2001). J Am Chem Soc.

[11] Kohlmann J, Braun T, Laubenstein R, Herrmann R (2017). Chem – Eur J.

[12] Hoffmann W, Langenhan J, Huhmann S, Moschner J, Chang R, Accorsi M, Seo J, Rademann J, Meijer G, Koksch B (2019). Angew Chem, Int Ed.

[13] Firooznia F, Gude C, Chan K, Fink C A, Qiao Y, Satoh Y, Marcopoulos N, Savage P, Beil M E, Bruseo C W (2001). Bioorg Med Chem Lett.

[14] Lee K-H, Lee H-Y, Slutsky M M, Anderson J T, Marsh E N G (2004). Biochemistry.

[15] Jäckel C, Koksch B (2005). Eur J Org Chem.

[16] Jäckel C, Salwiczek M, Koksch B (2006). Angew Chem, Int Ed.

[17] Salwiczek M, Samsonov S, Vagt T, Nyakatura E, Fleige E, Numata J, Cölfen H, Pisabarro M T, Koksch B (2009). Chem – Eur J.

[18] Budisa N, Wenger W, Wiltschi B (2010). Mol BioSyst.

[19] He T, Gershenson A, Eyles S J, Lee Y-J, Liu W R, Wang J, Gao J, Roberts M F (2015). J Biol Chem.

[20] Hammill J T, Miyake-Stoner S, Hazen J L, Jackson J C, Mehl R A (2007). Nat Protoc.

[21] Jackson J C, Hammill J T, Mehl R A (2007). J Am Chem Soc.

[22] Wiltschi B, Weber W, Fussenegger M (2012). Expressed Protein Modifications: Making Synthetic Proteins. Synthetic Gene Networks: Methods and Protocols.

[23] Furter R (1998). Protein Sci.

[24] Munier R, Cohen G N (1959). Biochim Biophys Acta.

[25] Moschner J, Stulberg V, Fernandes R, Huhmann S, Leppkes J, Koksch B (2019). Chem Rev.

[26] Andra K K (2015). Biochem Anal Biochem.

[27] Mallareddy J R, Tóth G, Fazakas C, Molnár J, Nagyőszi P, Lipkowski A W, Krizbai I A, Wilhelm I (2012). Chem Biol Drug Des.

[28] Mallareddy J R, Borics A, Keresztes A, Kövér K E, Tourwé D, Tóth G (2011). J Med Chem.

[29] Piepenbrink K H, Borbulevych O Y, Sommese R F, Clemens J, Armstrong K M, Desmond C, Do P, Baker B M (2009). Biochem J.

[30] Doubrovina E S, Doubrovin M M, Lee S, Shieh J-H, Heller G, Pamer E, O’Reilly R J (2004). Clin Cancer Res.

[31] Dominguez M A, Thornton K C, Melendez M G, Dupureur C M (2001). Proteins: Struct, Funct, Genet.

[32] Pace C J, Gao J (2013). Acc Chem Res.

[33] Dougherty D A (2013). Acc Chem Res.

[34] Oswald C L, Carrillo-Márquez T, Caggiano L, Jackson R F W (2008). Tetrahedron.

[35] Jackson R F W, Moore R J, Dexter C S, Elliott J, Mowbray C E (1998). J Org Chem.

[36] Ross A J, Lang H L, Jackson R F W (2010). J Org Chem.

[37] Ayoup M S, Cordes D B, Slawin A M Z, O'Hagan D (2015). Org Biomol Chem.

[38] Ayoup M S, Cordes D B, Slawin A M Z, O'Hagan D (2015). Beilstein J Org Chem.

[39] Liu S, Dockendorff C, Taylor S D (2001). Org Lett.

[40] Hill B, Ahmed V, Bates D, Taylor S D (2006). J Org Chem.

[41] Bosshard H R, Berger A (1973). Helv Chim Acta.

[42] Zheng H, Comeforo K, Gao J (2009). J Am Chem Soc.

[43] Li X, Xiao W, He G, Zheng W, Yu N, Tan M (2012). Colloids Surf, A.

[44] Kim D, Wang L, Beconi M, Eiermann G J, Fisher M H, He H, Hickey G J, Kowalchick J E, Leiting B, Lyons K (2005). J Med Chem.

[45] Setty S C, Horam S, Pasupuleti M, Haq W (2017). Int J Pept Res Ther.

[46] Castillo Meleán J, Ermert J, Coenen H H (2011). Org Biomol Chem.

[47] Vukelić S, Ushakov D B, Gilmore K, Koksch B, Seeberger P H (2015). Eur J Org Chem.

[48] Samet A V, Coughlin D J, Buchanan A C, Gakh A A (2002). Synth Commun.

[49] FILLER R, NOVAR H (1960). J Org Chem.

[50] Yu H, Richey R N, Stout J R, LaPack M A, Gu R, Khau V V, Frank S A, Ott J P, Miller R D, Carr M A (2008). Org Process Res Dev.

[51] Liu J, Deng X, Fitzgerald A E, Sales Z S, Venkatesan H, Mani N S (2011). Org Biomol Chem.

[52] Coenen H H, Franken K, Kling P, Stöcklin G (1988). Int J Radiat Appl Instrum, Part A.

[53] Ishiwata K, Ido T, Mejia A A, Ichihashi M, Mishima Y (1991). Int J Radiat Appl Instrum, Part A.

[54] Makaravage K J, Brooks A F, Mossine A V, Sanford M S, Scott P J H (2016). Org Lett.

[55] Geurink P P, Liu N, Spaans M P, Downey S L, van den Nieuwendijk A M C H, van der Marel G A, Kisselev A F, Florea B I, Overkleeft H S (2010). J Med Chem.

[56] Chiu H-P, Suzuki Y, Gullickson D, Ahmad R, Kokona B, Fairman R, Cheng R P (2006). J Am Chem Soc.

[57] Panella L, Aleixandre A M, Kruidhof G J, Robertus J, Feringa B L, de Vries J G, Minnaard A J (2006). J Org Chem.

[58] Verhoeven J, Hulpia F, Kersemans K, Bolcaen J, De Lombaerde S, Goeman J, Descamps B, Hallaert G, Van den Broecke C, Deblaere K (2019). Sci Rep.

[59] Ando M, Kuzuhara H (1990). Bull Chem Soc Jpn.

[60] Kukhar V P, Belokon Y N, Soloshonok V, Svistunova N Yu, Rozhenko A B, Kuz'mina N A (1993). Synthesis.

[61] Soloshonok V A, Belokon' Yu N, Kukhar' V P, Chernoglazova N I, Saporovskaya M B, Bakhmutov V I, Kolycheva M T, Belikov V M (1990). Russ Chem Bull.

[62] Drouet F, Noisier A F M, Harris C S, Furkert D P, Brimble M A (2014). Eur J Org Chem.

[63] Szymanski W, Wu B, Weiner B, de Wildeman S, Feringa B L, Janssen D B (2009). J Org Chem.

[64] Okuda K, Hirota T, Kingery D A, Nagasawa H (2009). J Org Chem.

[65] Davis F A, Srirajan V, Titus D D (1999). J Org Chem.

[66] Shi G-q, Zhao Z-y, Zhang X-b (1995). J Org Chem.

[67] Kollonitsch J, Marburg S, Perkins L (1975). J Org Chem.

[68] Tsushima T, Kawada K, Nishikawa J, Sato T, Tori K, Tsuji T, Misaki S (1984). J Org Chem.

[69] Tsushima T, Sato T, Tsuji T (1980). Tetrahedron Lett.

[70] Wade T N, Gaymard F, Guedj R (1979). Tetrahedron Lett.

[71] Bloom S, McCann M, Lectka T (2014). Org Lett.

[72] Egami H, Masuda S, Kawato Y, Hamashima Y (2018). Org Lett.

[73] Bume D D, Pitts C R, Jokhai R T, Lectka T (2016). Tetrahedron.

[74] Davies S G, Fletcher A M, Frost A B, Roberts P M, Thomson J E (2015). Org Lett.

[75] Davies S G, Fletcher A M, Frost A B, Roberts P M, Thomson J E (2014). Tetrahedron.

[76] Davies S G, Fletcher A M, Frost A B, Lee J A, Roberts P M, Thomson J E (2013). Tetrahedron.

[77] Zhang Q, Yin X-S, Chen K, Zhang S-Q, Shi B-F (2015). J Am Chem Soc.

[78] Zhu R-Y, Tanaka K, Li G-C, He J, Fu H-Y, Li S-H, Yu J-Q (2015). J Am Chem Soc.

[79] Meyer S D, Schreiber S L (1994). J Org Chem.

[80] Dess D B, Martin J C (1983). J Org Chem.

[81] Schlosser M, Brügger N, Schmidt W, Amrhein N (2004). Tetrahedron.

[82] Harada K, Fox S W (1964). Naturwissenschaften.

[83] Schlosser M, Soloshonok V A (1999). The Chemical and Physiological size of Fluorine. Enantiocontrolled Synthesis of Fluoro-organic Compounds: Stereochemical Challenges and Biomedicinal Targets.

[84] Wei Q, Ma Y, Li L, Liu Q, Liu Z, Liu G (2018). Org Lett.

[85] Vandenberghe S, Marsden P K (2015). Phys Med Biol.

[86] Martarello L, Schaffrath C, Deng H, Gee A D, Lockhart A, O'Hagan D (2003). J Labelled Compd Radiopharm.

[87] Deng H, Cobb S L, Gee A D, Lockhart A, Martarello L, McGlinchey R P, O'Hagan D, Onega M (2006). Chem Commun.

[88] Snyder H R, Reedy A J, Lennarz W J (1958). J Am Chem Soc.

[89] Ishiwata K, Ido T, Kawamura M, Kubota K, Ichihashi M, Mishima Y (1991). Int J Radiat Appl Instrum, Part B.

[90] Ishiwata K (2019). Ann Nucl Med.

[91] Ishiwata K, Ido T, Honda C, Kawamura M, Ichihashi M, Mishima Y (1992). Int J Radiat Appl Instrum, Part B.

[92] Chandra S, Kabalka G W, Lorey D R, Smith D R, Coderre J A (2002). Clin Cancer Res.

[93] Tscherpel C, Dunkl V, Ceccon G, Stoffels G, Judov N, Rapp M, Meyer P T, Kops E R, Ermert J, Fink G R (2017). Neuro-Oncology (Cary, NC, U S).

[94] Verhoeven J, Bolcaen J, Kersemans K, De Meulenaere V, Deblaere K, Kalala J, Van den Broecke C, Vanhove C, Goethals I, De Vos F (2017). Neuro-Oncology (Cary, NC, U S).

[95] Chiaravalloti A, Filippi L, Ricci M, Cimini A, Schillaci O (2019). Cancers.

[96] Moreau A, Febvey O, Mognetti T, Frappaz D, Kryza D (2019). Front Oncol.

[97] Lahoutte T, Caveliers V, Camargo S M, Franca R, Ramadan T, Veljkovic E, Mertens J, Bossuyt A, Verrey F (2004). J Nucl Med.

[98] Bolcaen J, Descamps B, Deblaere K, Boterberg T, De Vos Pharm F, Kalala J-P, Van den Broecke C, Decrock E, Leybaert L, Vanhove C (2015). Nucl Med Biol.

[99] Feral C C, Tissot F S, Tosello L, Fakhry N, Sebag F, Pacak K, Taïeb D (2017). Eur J Nucl Med Mol Imaging.

[100] Sun A, Liu X, Tang G (2018). Front Chem (Lausanne, Switz).

[101] Chiotellis A, Müller Herde A, Rössler S L, Brekalo A, Gedeonova E, Mu L, Keller C, Schibli R, Krämer S D, Ametamey S M (2016). J Med Chem.

[102] Strese S (2015). Anticancer Activity of Melflufen: Preclinical Studies of a Novel Peptidase-Potentiated Alkylator.

[103] Carlier C, Strese S, Viktorsson K, Velander E, Nygren P, Uustalu M, Juntti T, Lewensohn R, Larsson R, Spira J (2016). Oncotarget.

[104] Ormerod D, Willemsens B, Mermans R, Langens J, Winderickx G, Kalindjian S B, Buck I M, McDonald I M (2005). Org Process Res Dev.

[105] Chau I, Cunningham D, Russell C, Norman A R, Kurzawinski T, Harper P, Harrison P, Middleton G, Daniels F, Hickish T (2006). Br J Cancer.

[106] Harper E A, Roberts S P, Kalindjian S B (2007). Br J Pharmacol.

[107] McDonald I M, Black J W, Buck I M, Dunstone D J, Griffin E P, Harper E A, Hull R A D, Kalindjian S B, Lilley E J, Linney I D (2007). J Med Chem.

[108] Harper E A, Mitchell E A, Griffin E P, Kalindjian S B (2008). Eur J Pharmacol.

[109] Mei H, Han J, Klika K D, Izawa K, Sato T, Meanwell N A, Soloshonok V A (2020). Eur J Med Chem.

[110] Roberts S, Griffin E, Harper E, Hull R, Kalindjian S, Lilley E, Kotecha A, Shankley N, Sykes D, Watt G (2002). Pharmacologist.

[111] Herranz R (2003). Med Res Rev.

[112] Berna M J, Tapia J A, Sancho V, Jensen R T (2007). Curr Opin Pharmacol.

[113] Pippel M, Boyce K, Venkatesan H, Phuong V K, Yan W, Barrett T D, Lagaud G, Li L, Morton M F, Prendergast C (2009). Bioorg Med Chem Lett.

[114] Herman G A, Stevens C, Van Dyck K, Bergman A, Yi B, De Smet M, Snyder K, Hilliard D, Tanen M, Tanaka W (2005). Clin Pharmacol Ther (Hoboken, NJ, U S).

[115] Kushwaha R N, Haq W, Katti S (2014). Chem Biol Interface.

[116] Baksh S N, McAdams-DeMarco M, Segal J B, Alexander G C (2018). Pharmacoepidemiol Drug Saf.

[117] Kaihong Y, Piaoyang S (2013). Pharmaceutical composition for treatment of type 2 diabetes. U.S. Patent.

[118] (2020). Drugbank, Retagliptin.

[119] Notte G T (2014). Annu Rep Med Chem.

[120] Yong X, Hu T, Feng S, Du X, Shi H, Feng W (2015). Trop J Pharm Res.

[121] McCormack P L (2015). Drugs.

[122] Flick A C, Ding H X, Leverett C A, Kyne R E, Liu K K-C, Fink S J, O’Donnell C J (2017). J Med Chem.

[123] Oh J, Kim A H, Lee S, Cho H, Kim Y S, Bahng M Y, Yoon S H, Cho J-Y, Jang I-J, Yu K-S (2017). Diabetes Obes Metab.

[124] Zhao W (2016). Handbook for Chemical Process Research and Development.

[125] Fraser G L, Hoveyda H R, Tannenbaum G S (2008). Endocrinology.

[126] Lasseter K C, Shaughnessy L, Cummings D, Pezzullo J C, Wargin W, Gagnon R, Oliva J, Kosutic G (2008). J Clin Pharmacol.

[127] Hoveyda H R, Marsault E, Gagnon R, Mathieu A P, Vézina M, Landry A, Wang Z, Benakli K, Beaubien S, Saint-Louis C (2011). J Med Chem.

[128] Gallwitz B (2011). Drugs.

[129] Trujillo J M, Nuffer W (2014). Pharmacotherapy.

[130] Gallwitz B, Gough S (2016). Glucagon-like peptide-1 receptor agonists. Handbook of Incretin-based Therapies in Type 2 Diabetes.

[131] Theile J W, Cummins T R (2011). Front Pharmacol.

[132] Hoyt S B, London C, Abbadie C, Felix J P, Garcia M L, Jochnowitz N, Karanam B V, Li X, Lyons K A, McGowan E (2013). Bioorg Med Chem Lett.

[133] Hoyt Scott B, London C, Ok D, Parsons W H (2011). Benzaepinones as sodium channel blockers. U.S. Patent.

[134] Hoyt S B, London C, Gorin D, Wyvratt M J, Fisher M H, Abbadie C, Felix J P, Garcia M L, Li X, Lyons K A (2007). Bioorg Med Chem Lett.

[135] Hoyt S B, London C, Ok H, Gonzalez E, Duffy J L, Abbadie C, Dean B, Felix J P, Garcia M L, Jochnowitz N (2007). Bioorg Med Chem Lett.

